# Blooming of a microbial community in an Ediacaran extreme volcanic lake system

**DOI:** 10.1038/s41598-023-36031-5

**Published:** 2023-06-05

**Authors:** Ibtissam Chraiki, Ernest Chi Fru, Andrea Somogyi, El Hafid Bouougri, Olabode Bankole, Mohamed Ghnahalla, Abderrazak El Albani

**Affiliations:** 1 Department of Geology, Faculty of Sciences-Semlalia, Cadi Ayyad University, Marrakesh, Morocco; 2 Centre for Geobiology and Geochemistry, College of Physical and Engineering Sciences, School of Earth and Environmental Sciences, Cardiff University, Cardiff, UK; 3 Nanoscopium Beamline Synchrotron Soleil, Saint-Aubin, 91192 Gif-sur-Yvette, France; 4 CNRS IC2MP UMR 7285, University of Poitiers, Poitiers, France

**Keywords:** Biogeochemistry, Planetary science

## Abstract

Ancient aquatic sediments are critical archives for studying early microbial life and the types of environments in which they thrived. The recently characterized Amane Tazgart microbialites in the Anti-Atlas, Morocco, are a rare and well-preserved non-marine deposit that evolved in an alkaline volcanic lake setting during the Ediacaran Period. A multiproxy geochemical toolbox reveals evidence pointing to spatio-temporal ecosystem organization and succession related to changing lake water chemistry. This is marked by secular transition from a cold/dry climate, hypersaline alkaline thermophilic and anoxic–oxic community, to a stable state warm/wet climate fully oxygenated fresh to brackish water ecosystem, predominated by oxygenic stromatolites. Extreme dissolved Arsenic concentrations suggest that these polyextremophiles required robust detoxification mechanisms to circumvent arsenic toxicity and phosphate deficiency. We propose that self-sustaining and versatile anoxic to oxic microbial ecosystems thrived in aquatic continental settings during the Ediacaran Period, when complex life co-evolved with a rise in atmospheric oxygen content.

## Introduction

Modern aquatic microbial ecosystems are known to produce distinct spatio-temporal communities along geochemical gradients^1–3^ that tend to precipitate diagnostic biogenic minerals such as carbonates, iron oxides, and pyrite^3–12^. Geochemical characterization of these microbial habitats ties shale-normalized rare earth element (REE), yttrium (Y), and redox-sensitive trace element distribution to prevailing environmental redox conditions, chemistry, and mineral deposition^11,13^. Particularly, REE patterns normalized to Post Archean Australian Shales (PAAS), marked by light REE depletion, negative cerium (Ce) and slightly positive lanthanum (La) anomalies, and high Y to holmium (Y/Ho) ratios, collectively reflect oxygenated modern seawater mass conditions^14^; and in certain instances, non-marine aquatic ecosystems influenced by elevated alkalinity^15^. In fact, the firm connection of REE + Y (REY) fractionation to dissolved carbonate ion concentration, serves as a valuable tool for reconstructing alkalinity trends, especially in restricted aquatic ecosystems^15^.

On the other hand, the relative abundance of europium (Eu) is often associated with hydrothermal fluid enrichment, feldspars, and diagenesis^16,17^. While negative Eu anomalies may represent the original composition of the aquatic environment from which limestones formed^16^, and La, gadolinium (Gd), and Y anomalies, mineral complexation phenomena unique to the hydrosphere^18^. Hence, authentic, uncontaminated marine, estuarine, and lacustrine water chemistries are often deduced from REY systematics.

Various geochemical palaeosalinity proxies utilizing trace elemental ratios in bulk mudstones are among the most popular. They are founded on the observation that certain non-redox-sensitive elements show relatively higher concentrations in seawater such as B, Y, and Sr, or freshwater, like Ga, Ho, and Ba, on a salinity-normalized basis^19–24^. Elemental cross plots allow estimation of expected salinity baselines for each proxy: (1) B/Ga > 6 is typical of marine facies, 3–6 brackishness, and < 3 freshwater^20,23^; (2) Sr/Ba is > 0.5 characterizes marine deposits, 0.2–0.5 brackishness, and < 0.2 freshwater^20^; and (3) B/K > 40 μg/g distinguishes marine from brackish/freshwater sediments^22,25^. Furthermore, several geochemical parameters such as Ni/Co, Cu/Zn, V/Cr, V/(V + Ni), U, U/Th, authigenic trace elements, and Ce/Ce*, are used to provide estimates for paleo-redox depositional conditions^26–28^. However^29^, in the absence of living biomass and the associated biosignatures that enable comparative characterization of modern aquatic microbial communities, very little evidence exists directly linking microbial community succession patterns to long-term environmental drivers in the Proterozoic biosphere. This has led to considerable difficulties in our ability to unambiguously characterize and tie microbial processes to shifting geochemical gradients, environmental disturbances, and geodynamics in deep-time ecosystems, further weakening attempts at coupling biological evolution to evolving early Earth surface chemistry.

Here, we combine high-resolution multiproxy geochemistry to a sequence of well-preserved microbialites in a continuous sedimentary profile of an Ediacaran alkaline lake ecosystem from Morocco's Anti-Atlas^30^, to demonstrate a strong link between changing environmental chemistry and the assemblage, organization, and succession of a rare extremophilic microbial community. We identify and characterize the discrete biogeochemical intervals that drove their spatial and temporal differentiation into distinct assemblages.

## Results

### Whole rock geochemistry

Major elemental composition of the Amane Tazgart microbialites shows two distinct patterns defined by their carbonate and epiclastic chemistries (Supplementary Table 1). The concentrations of major elements generally change across the succession, with major elements correlating positively with Al_2_O_3_ in the carbonates, except for MnO and a negative correlation for CaO (Supplementary Fig. 1). Fe_2_O_3_, Na_2_O, TiO_2_, and P_2_O_5_ equally correlate positively with Al_2_O_3_ in the epiclastic samples, while the rest show no relationship (Supplementary Fig. 1). In the carbonates, P_2_O_5_ content is consistently below the detection limit of 0.1% but above this threshold in the epiclastic samples. The concentrations of As, Ba, Cr, Cu, Pb, Sb, U, V, and Zr are significantly high, while Mo is below the detection limit of 0.5 ppm in most of the carbonates and epiclastic samples, except for four samples (AT2, AT3-C, AT12-S, and AT27), which had values ranging from 0.63 to 15.1 ppm (Supplementary Table 2). The As content decreased upward in the section with a general variation from 108 to 277 ppm, averaging 189 ppm in the carbonates and 22.7–77.2 ppm with an average of 49.62 ppm in the epiclastic deposits (Supplementary Table 2). The trace element systematics are detailed in Supplementary Note 1.

**Figure 1:**
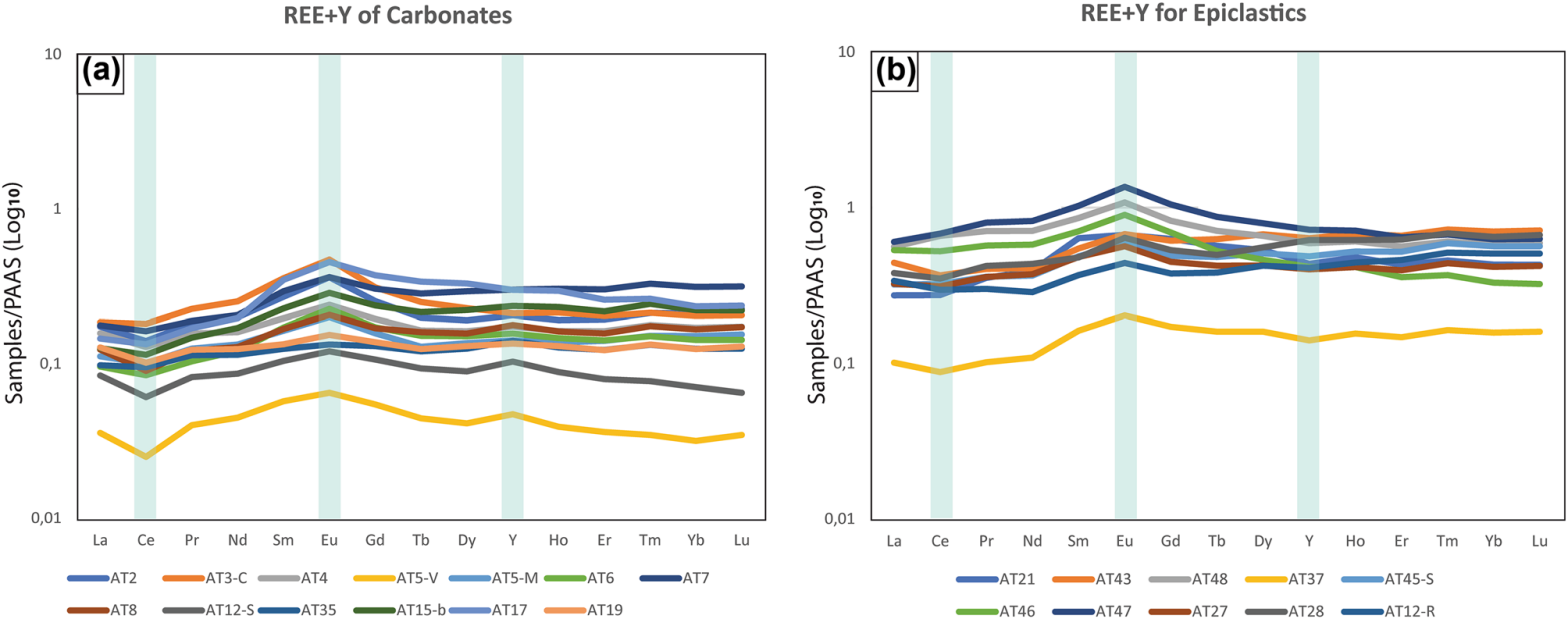
Rare Earth Element and Yttrium spectra (REE + Y). (**a**) REY spectra of carbonates. (**b**) REY spectra of Epiclastic deposits.

The epiclastic microbialites are typically more enriched in REY than the carbonates compared to PAAS (Fig. 1). Conversely, the carbonate-dominated samples are more enriched in LREE relative to the HREE compared to the epiclastic-dominated samples (Supplementary Note 2) (Fig. 1). Generally, the sampled sequence exhibits positive Eu anomaly (Eu/Eu*) values, varying from 1.04 to 1.41 with a mean of 1.22, and negative Ce anomalies (Ce/Ce*) range from 0.69 to 0.94 with an average of 0.87 (Supplementary Table 3). Y/Ho ratio averages 27.75 for all samples, with values being mostly > 28 and typically associated with the carbonates, except for four samples (AT3C, AT7, AT15-b, and AT17) showing values similar to those recorded in the epiclastic microbialites (Supplementary Table 3). ΣREY varies from 7.63 to 44.47 in the carbonates, increasing to > 23.21 and up to 152.71 in the epiclastic deposits (Supplementary Table 4).

### Arsenic intercorrelation with other elements

**Figure 2:**
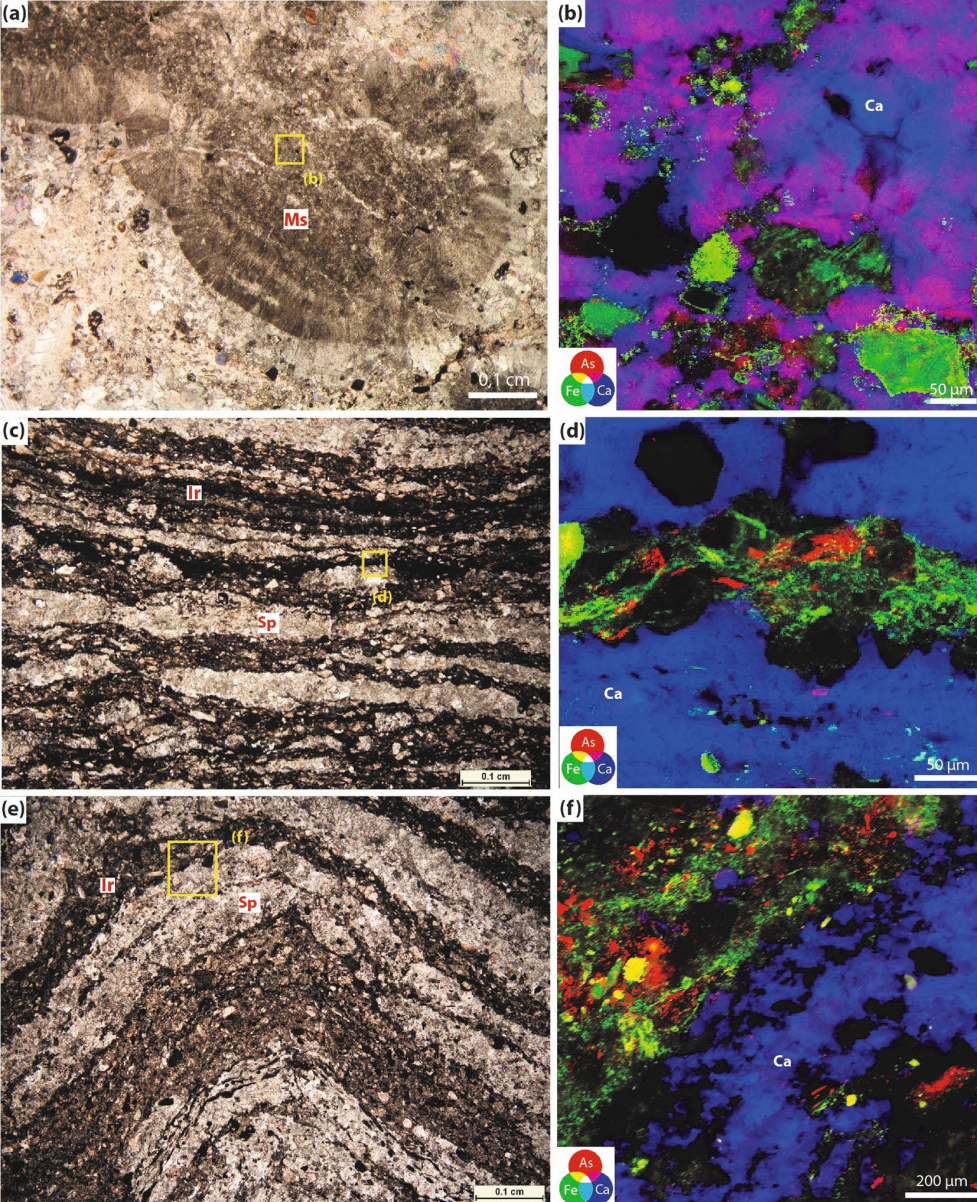
(**a**) Thin section photomicrograph of a thrombolite showing a mesoclot characterized by a micritic core (Ms). (**b**) RGB image of the distribution of As (red), Fe (green), and Ca (blue) in the area indicated by the yellow square in (**a**), showing the diffused distribution of As within calcite. (**c**) Thin section photomicrograph of a stromatolite showing an alternation between iron microbial crusts (Ir) and sparitic crusts (Sp). (**d**) RGB image of the distribution of As, Fe, and Ca in the area indicated by the yellow square in (c), showing the condensed concentration of As within iron microbial crust. (**e**) Thin section photomicrograph of a stromatolite composed of the alternation of grain-sized microbial laminae (Gs) and sparitic crusts (Sp), (**f**) RGB images of the distribution of As, Fe, and Ca of grain-sized microbial laminae and a sparitic crust expressing the enrichment of grain-sized laminae in irons and arsenic.

**Figure 3:**
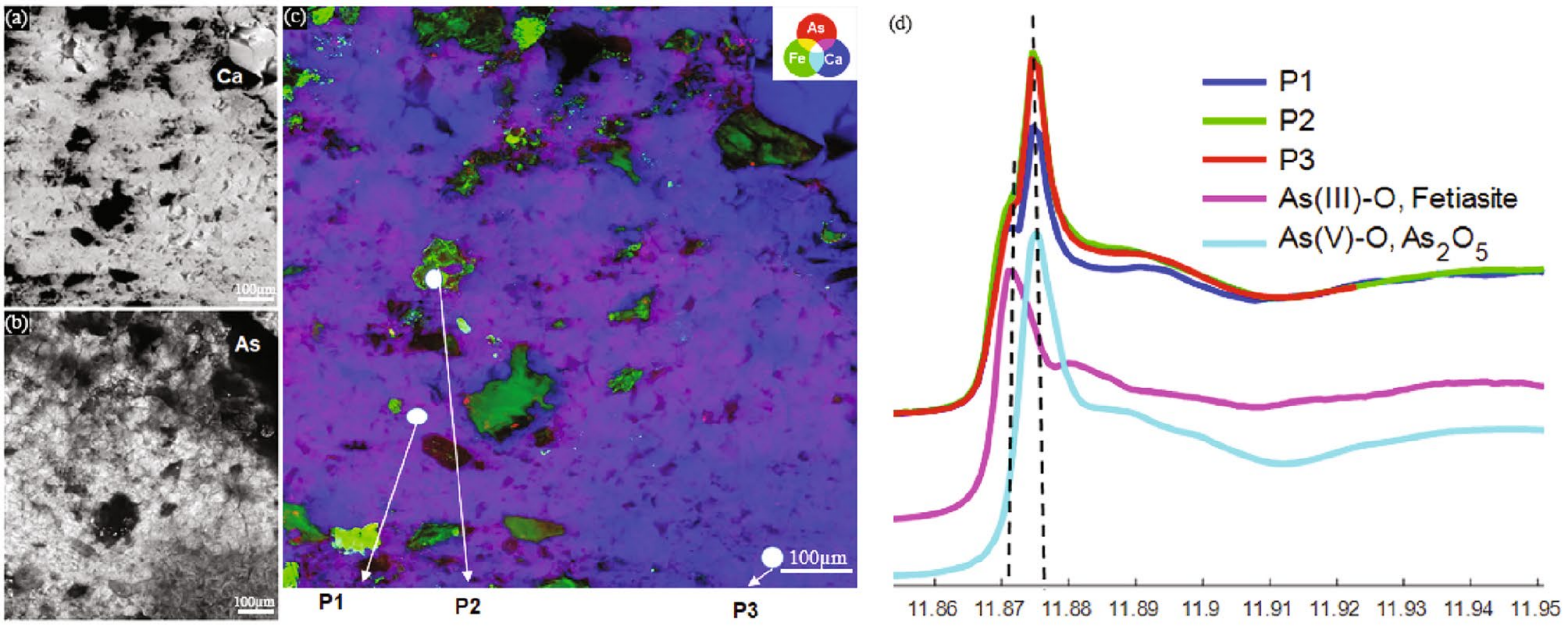
(**a**) and (**b**) As and Ca distribution in a carbonate sample, respectively, in (**c**). Lighter colors indicate higher XRF intensities (higher elemental concentrations). The typical crystal shape of the nano-/micro-carbonates reflected in the carbonate distribution indicates that As is distributed homogenously within these phases. (**c**) RGB image of the distribution of As, Fe and Ca showing the points where XANES spectra were measured. (**d**) XANES spectra at the As k-edge were obtained in three representative points from (**c**).

**Figure 4:**
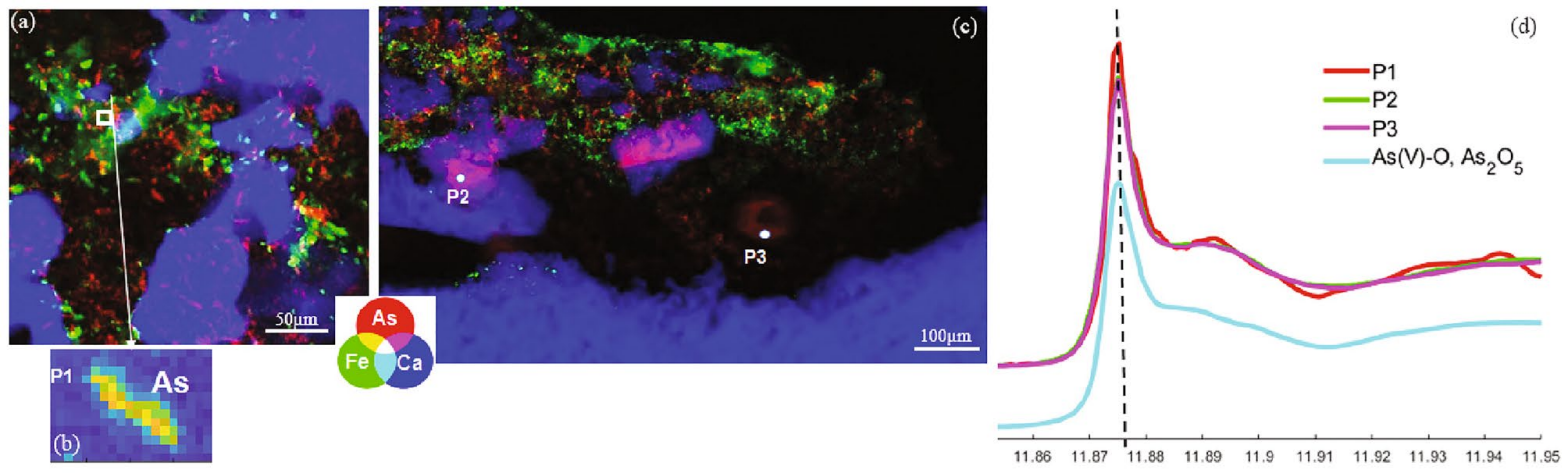
(**a**) RGB of As, Fe, and Ca distribution in a carbonate sample showing the association of As within iron, with an inset view of the disseminated distribution of As marked by a white square for (**b**), corresponding to point 1 of the XANES spectra. (**c**) RGB image of As, Fe, and Ca distribution measured in a different location of the same sample as (**a**), showing two points where XANES spectra were performed. (**d**) XANES spectra at the As K-edge were obtained in three representative points from both (**a**) and (**c**).

The concentration of As in all the samples is considerably high, but more enriched in the carbonate than in the epiclastic rocks (Supplementary Note 1, Supplementary Table 5). To evaluate the distribution, location, and sinks of As within the microbialites, nanoscale Synchrotron radiation X-ray micro-Fluorescence (SR-XRF) imaging was performed on several representative carbonate and epiclastic samples, distinguished by fabric similarity and dissimilarity. The investigated samples included iron- and carbonate-rich areas, corresponding to the main microbial products. XRF imaging shows that As is either colocalized with Ca or can be found within iron-rich microbial laminae (Fig. 2). In the carbonate microbial deposits, thrombolites, and composite microbialites, As is mainly concentrated in the peloidal micritic areas within mesoclots (Fig. 2a,b) but rarely associated with the iron-rich mineral phases that are generally detrital in the case of the thrombolitic and composite microbialites. In contrast, stromatolitic fabrics in both the carbonates and epiclastic rocks tend to concentrate As within the iron-rich microbial laminae but not in sparitic laminae (Fig. 2c–f). Strikingly, As can be found within nano-and micro-crystalline carbonate phases, as shown by the pinkish magenta areas in the RGB images (Fig. 2b), corresponding to Ca (blue)-As (red) colocalization. The gray scale image of the As and Ca distribution in Fig. 3a,b shows that the distribution of As reflects the typical crystal shape of the nano-/micro-carbonates, indicating that As is distributed homogenously within these phases. On the other hand, in the iron-rich microbial laminae the distribution of As is heterogenous at the nanometer and micrometer scale containing micrometer-sized As-rich areas and elongated fiber-like structures (Fig. 2f–d). Raman spectroscopy on different types of iron-rich laminae in the epiclastic stromatolites show that the iron-rich laminae are composed of hematite (Supplementary Fig. 2), as is the bulk iron mineralogy of the studied section^32^. As speciation in the microbial products by Nano-XANES analysis of two different carbonate samples (Figs. 3c, 4a–c), revealed the presence of As(III-O) and As(V–O) within calcite deposits (Fig. 3d) and predominantly As(V–O) in the iron oxides (Fig. 4d).

### Chemical weathering and sediment provenance

**Figure 5:**
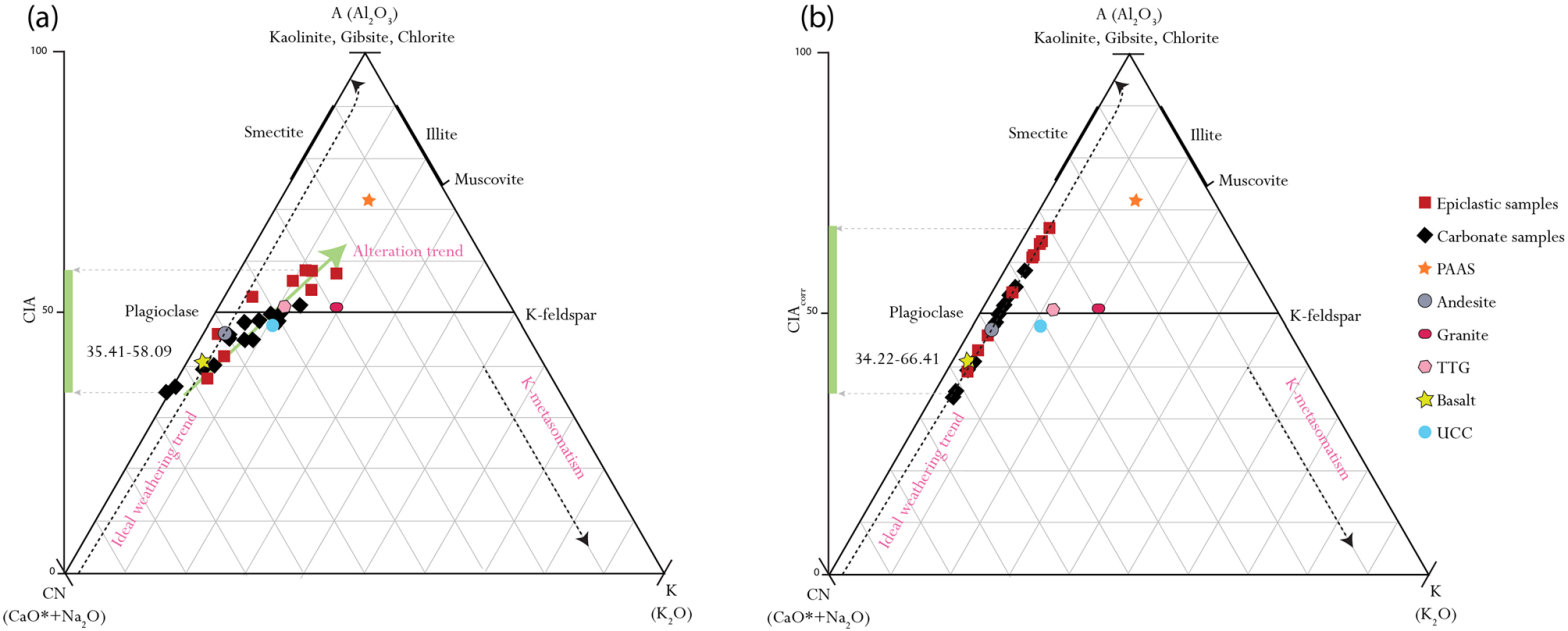
(**a**) A-CN-K ternary diagram with uncorrected data. (**b**) A-CN-K ternary diagram with corrected data. CIA–Chemical Index of Alteration, CIAcorr–Corrected CIA, A–Al_2_O_3_, CN–CaO* + Na_2_O, K–K_2_O (all in molar proportions), CaO*–CaO incorporated in the silicate fraction of the sample. Note: average Upper Continental Crust (UCC; Taylor and McLennan^87^); Post-Archean average Australian Shale (PAAS: Taylor and McLennan^87^); Andesite; Granite; TTG and Basalt (Condie^88^).

The projection of the Amane Tazgart samples in the A-CN-K ternary diagram (Fig. 5a) shows CIA values ranging from 35.41 to 51.61 and 38.24 to 58.08 in the carbonates and epiclastic deposits, respectively. Most of the samples deviate from the ideal weathering trend on the A-CN-K diagram, with the tendency to be projected toward the A-K axis suggesting pervasive k-metasomatism may have affected bulk sample composition^31,32^. The K-corrected CIA values (CIA_corre_) range from 34.22 to 58.22 with an average of 47.17 in the carbonates, and from 38.93 to 66 with an average of 55.25 in the epiclastic rocks. They follow the ideal weathering trend (Fig. 5b) and project the samples to a potential andesite protolith. In support of this observation, Th/Sc versus La/Sc discriminant diagrams used to infer parent rock source^33,34^ (Supplementary Fig. 3), placed the samples in the mafic field, close to an andesite affinity (Supplementary Fig. 3), thus confirming andesite as the likely dominant parent rock source of our samples. This observation is supported by the prevalence of andesitic rocks underlying and overlying the Amane Tazgart succession^35^ and around the studied lake.

## Discussion

The Eu^3+^/Eu^2+^ equilibrium in aqueous solutions is viewed as broadly dependent on pressure, pH, speciation, and in particular, temperature, according to theoretical and experimental considerations^36,37^. Eu^3+^ dominates in oxidized conditions near the Earth’s surface, except in highly reducing and alkaline settings, whereas Eu^2+^ dominates at temperatures > 250 °C^37^, where REY_N_ patterns with characteristic positive Eu anomalies are produced^38^. Therefore, Eu is enriched in Ca-rich and early-magmatic minerals such as plagioclase feldspar^39^. We produced cross plots of Eu/Eu*, Al, and Y/Ho ratio to discriminate the detrital flux versus hydrothermal origin of the positive Eu anomaly in the studied samples (Supplementary Fig. 4). There is no considerable correlation between Al and Eu/Eu* in our samples (Supplementary Fig. 4a). All the samples plot predominantly in the zone of strong hydrothermal influence with Y/Ho > 25 and Eu/Eu* > 1 (Supplementary Fig. 4b). Together, these observations suggest that the Eu/Eu* anomaly is not overprinted by detrital input and probably derived from hydrothermal fluids. Moreover, the enrichment of light rare earth elements (LREE) over (HREE) in several samples (Supplementary Fig. 5) is consistent with the influence of high temperature hydrothermal fluids characterized by LREE enrichment and positive Eu anomalies^40^. For comparison, we used a cherty sample from Archean Josefsdal Chert (3.5–3.33 Ga)^41^, as a reference of microbialites deposited in a restricted shallow marine environment, strongly influenced by hydrothermal activity^41,42^. The average of carbonate REE + Y patterns displays Josefsdal Chert-like REE + Y trends (Supplementary Fig. 6), supporting the view that hydrothermal fluids were a factor. Although the epiclastic sediments have the same REE + Y pattern, their Eu/Eu* and Ce/Ce* compositions are more attenuated, indicating that hydrothermal fluids have less of an impact on them.

Salinity is an essential chemical feature of water masses that critically regulates homeostatic balance in aquatic biological systems^25^. It serves as a crucial modulator of microbial biomass volume and biodiversity in alkaline lakes known to possess generally high and variable salinity levels^43^. Although salinity is challenging to evaluate compared to various palaeoenvironmental parameters in the sedimentary rock record, some elements, such as Y, Ho, B, Ga, Sr, and Ba have been successfully applied as geochemical proxies in the reconstruction of paleoenvironmental salinity condition^20–22,44,45^. In this study, data from the Triassic Zhangjiatan formation^46^, interpreted as deposited in a fresh lake ecosystem^20,46^, were employed as a paleosalinity reference.

**Figure 6:**
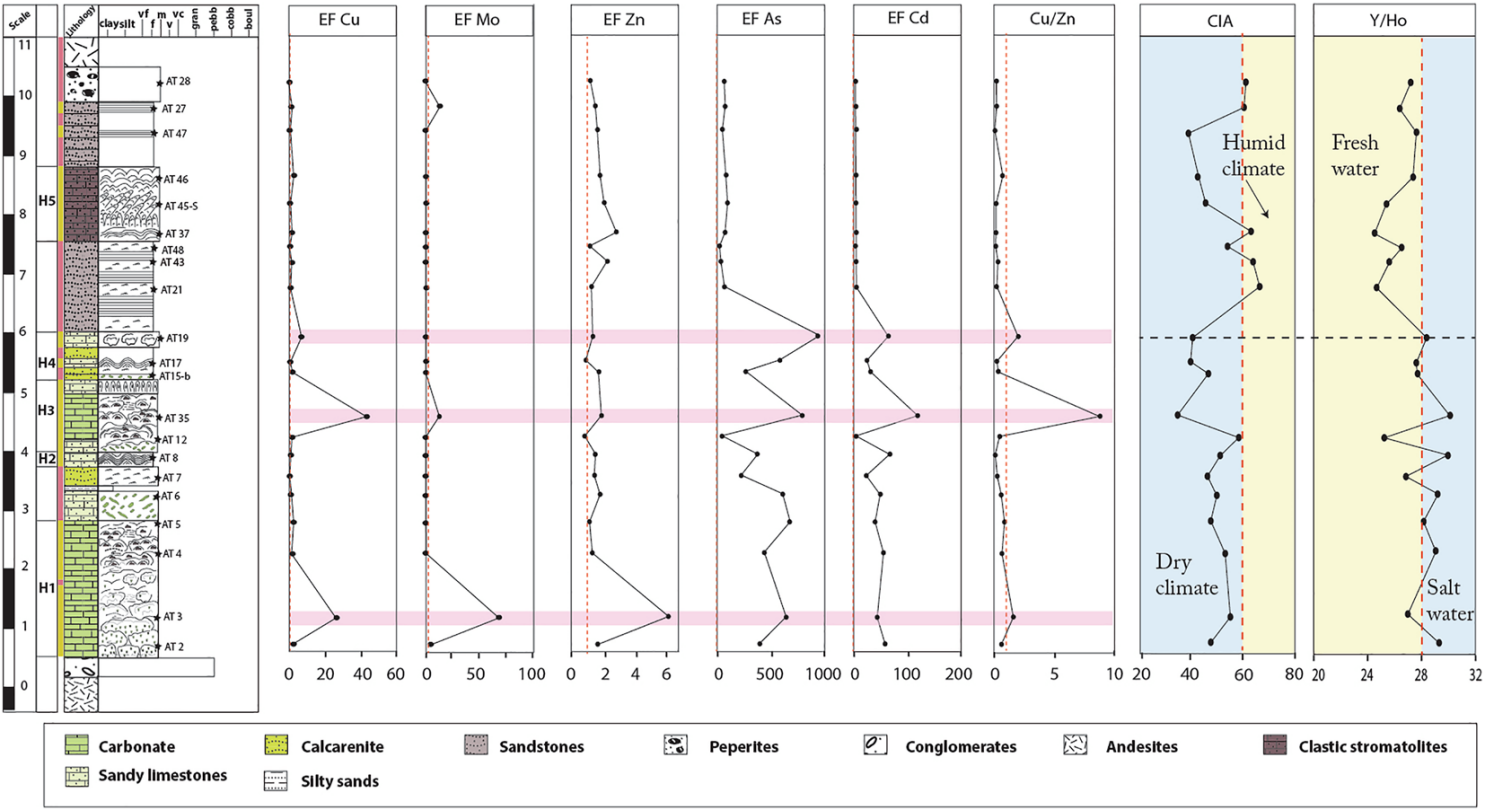
Stratigraphic profile of enrichment factors (EF) of selected redox trace elements, the chemical index of alteration (CIA), and the Y/Ho ratios as a salinity proxy of Amane Tazgart succession. (EF of Co, Cr, Ni, Th, U, V, and Zn, were calculated relative to the average shale PAAS (Taylor and McLennan^87^), and As relative to the UCC (McLennan^89^). The lithostratigraphic column is modified from Chraiki et al.^31^.

Y/Ho ratios in chemical sediments such as carbonates are elevated when deposited in saline waters due to the preferential stabilization of Y by anionic salts, resulting in a positive correlation between Y/Ho ratios and salinity^45^. Our carbonates exhibit an average Y concentration of 5.82 ppm with a tendency to vary from 3.79 to 11.1 ppm, and average Ho content of 0.21 ppm being in the range of 0.127–0.439 ppm. This yielded an average Y/Ho ratio of 28.19 ppm with a spread of 0.07–25.24 ppm (Supplementary Table 3). The epiclastic samples contain an average Y content of 13.3 ppm distributed between 3.79 and 19.5 ppm, being 0.5 ppm for Ho and spanning 0.154–0.705 ppm. This produced a Y/Ho ratio of 26.14 ppm with a spread of 24.57–27.61 (Supplementary Table 3). The difference in the average Y/Ho ratio of up to 2.05 ppm from the carbonates to the epiclastic deposit is consistent with salinity decreasing toward the top of the section. Thus, saltwater conditions are suggested to have been generally prevalent when the carbonates deposited, comparable to seawater conditions. The consistency of the data with the typical high salinity of modern alkaline lakes^17,45^, points to a temporal transition from saline towards a freshwater ecosystem from the underlying carbonates, up into the stratigraphically younger epiclastic deposit (Fig. 6). There are no Y or Ho data for the Zhangjiatan Formation^46^.

**Figure 7:**
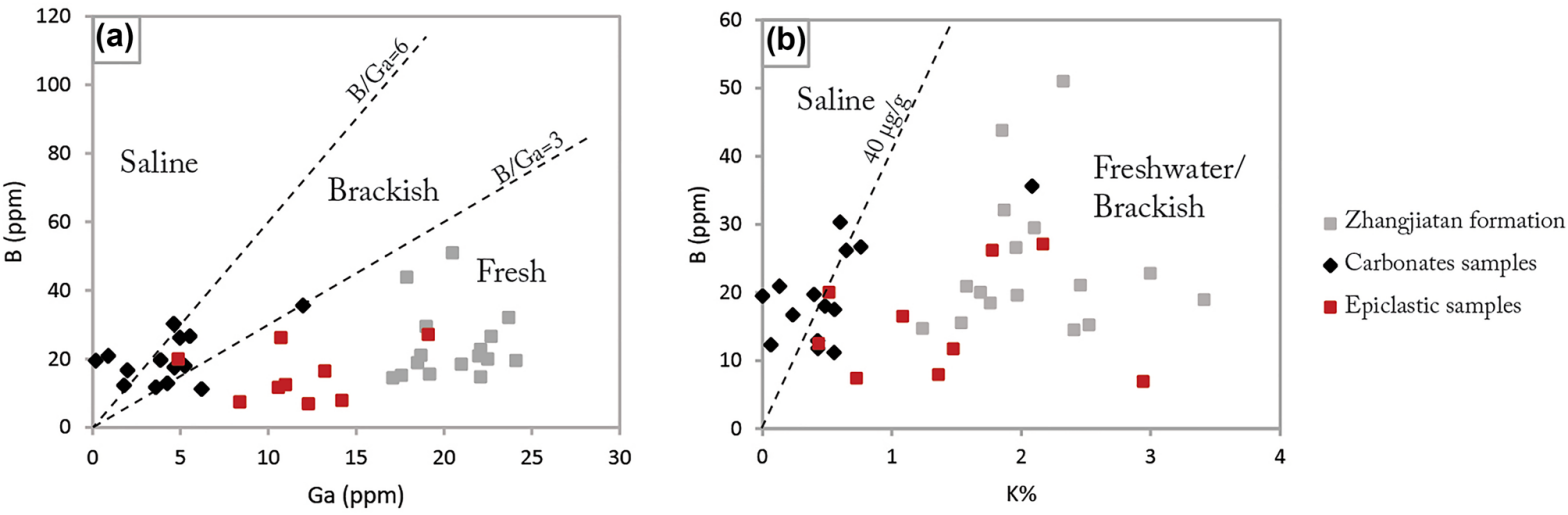
Salinity proxies for Amane Tazgart samples. (**a**) Biplots of B versus Ga. (**b**) B versus K (%).

Another proposed bulk mudstone paleosalinity proxy is B versus Ga plots, widely applied in different settings^20,21,25^, on the assumption that high values of this index are associated with the marine-type origin of sediments, owing to a higher concentration of B in seawater than in freshwater, whereas Ga is unaffected by salinity. Consequently, B/Ga ratios are < 3 in freshwater, 3–6 in brackish, and > 6 in marine-type facies^20^. The B-Ga biplots for the carbonate and epiclastic samples place the carbonates in the saline and brackish fields and the epiclastic samples in the freshwater field (Fig. 7a), consistent with the Y/Ho results. Comparatively, samples of the Triassic Zhangjiatan Formation plot in the freshwater field, in agreement with the findings of Li et al.^46^ and Wei, et al.^20^.

These observations are further supported by the important B/K ratio that has been effectively applied as a salinity proxy on modern sediments^22,47^ and on deeply buried Paleozoic and Ediacaran fossils^22^. The B/K ratios of 40 μg/g distinguish marine from non-marine salinities, including riverine and lacustrine deposits, but do not discriminate between brackish and freshwater salinities^22,48,49^. Our compiled dataset reveals B/K ratios of 17.09–189.96 for carbonates, 2.34–38.8 for epiclastic deposits, and 5.54–23.66 for the lacustrine Zhangjiatan Formation (Supplementary Table 6), suggesting overall that the carbonates were deposited in higher salinity waters, compared to the epiclastic lithologies and the Zhangjiatan Formation that plot in the brackish-freshwater field (Fig. 7b), which agree with the conclusions drawn from the Y/Ho and B/Ga ratios.

Paleosalinity reconstruction based on Sr/Ba ratios is frequently used to differentiate between freshwater, brackish, and full marine facies^20,21,46^. However, high sediment carbonate content may impact the reliability of this proxy^20^, as carbonate can raise bulk Sr/Ba ratios in comparison to the clay mineral fraction. The Amane Tazgart samples include up to 48% carbonates, which renders the Sr/Ba ratio unsuitable as a paleosalinity proxy in this case.

Chemical weathering on the continents is assumed to be constrained mainly by variations in atmospheric temperature and humidity. Thus the chemical index of alteration (CIA) was proposed by Nesbitt and Young^32^ as a proxy for the degree of chemical weathering of soils and sediments, with important inferences for paleoclimate reconstruction^50,51^. In arid and polar climates, chemical weathering intensity is commonly weak due to cold temperatures and limited precipitation^52^. Physical weathering or mechanical erosion, for example, due to salt wedging, the formation and thawing of ice, and the grinding of rocks by moving icesheets, prevail over chemical weathering in these settings, resulting in the formation of debris with the same chemical composition as the parent rocks^50^. On the other hand, chemical reactions between rocks and a solvent, resulting in the selective removal of mobile cations during chemical weathering, are facilitated by warmer and wetter environment conditions. Chemical sediments, therefore, tend to assume chemical compositions that are dissimilar to the parent rocks from which they were sourced and are characterized by higher CIA values.

The K_2_O content of rocks can be impacted by K-metasomatism, a post-depositional process in which K^+^ is absorbed from burial fluids by secondary mineral phases in highly altered rocks^31,53^. The extent of K enrichment can be ascertained from the A-CN-K plot, through deviations from the theoretical alteration trend that must be parallel to the A-CN axis^31,50^. Any deviation toward the A-K axis represents K addition by K-metasomatism^31,50,51^.

Chemical weathering significantly affects the mineralogy and major elemental geochemistry of siliciclastic sediments^32,54,55^. Quantitative parameters, including the CIA^32^, are therefore theoretically useful in evaluating the degree of chemical weathering. High CIA values generally > 60 reflect the elimination of labile cations (e.g. Ca^2+^, Na^+^, K^+^) compared to stable residual constituents of Al^3+^, and Ti^4+^ during weathering^31,32,50^. Inversely low CIA values being generally < 60, reflect the virtual absence of chemical weathering and thus may indicate cool and/or arid conditions.

The CIA record of the Amane Tazgart succession shows systematic changes closely related to climatic fluctuations (Fig. 6), potentially linked to the end of the preceding Gaskiers glaciations 580–581 million years ago^56^. While the K-corrected (CIA_corr_) for the carbonate deposits presents persistently low values with a mean of 47.17, implying weak chemical weathering typical of a dry and cold climate, the epiclastic deposits show an increasing CIA_corr_ trend, with values reaching up to 66.42 suggesting warmer and possibly wetter climatic conditions (Figs. 5b, 6, Supplementary Table 7). The predicted arid and wetter climatic conditions in which the carbonates and epiclastic lithologies are deposited, are well correlated with the high salinity lake setting required for the formation of the carbonate microbialites and the brackish to freshwater epiclastic microbialite depositional conditions, respectively (Fig. 6). The preceding cold/dry climatic conditions are consistent with the requirement for intense evaporation to increase water salinity levels during the deposition of carbonate-rich microbialites^57^. These results are similar to actual modern extreme alkaline lake system conditions where the growth and deposition of carbonate microbialites occur mostly in the dry season linked to higher water evaporation rates^57^.

The REE composition of carbonates, particularly for microbial and reef carbonates, provides insights into paleo-environmental features^58,59^. For instance, the Ce/Ce* values of carbonates highlight redox depositional conditions. Nevertheless, Ce anomalies of original aquatic carbonates could be influenced by diagenesis^39,58^ and detritus^58^.

Shields and Stille^39^ state that diagenetic exchange after deposition would influence REE patterns towards becoming progressively more Ce-enriched and Eu-depleted, with decreased Dy_N_/Sm_N_ ratios. Such diagenetic exchange would blur or decrease primary features associated with REE distribution patterns and might be recognized by negative correlations between Ce/Ce* versus Eu/Eu* and Dy_N_/Sm_N_^39^. All samples in this study exhibit small positive Eu anomalies that vary from 1.04 to 1.41 and show a positive correlation with Ce anomalies calculated by using the equation of Lawrence et al.^19^. The negative correlations between Ce/Ce* versus Eu/Eu* and Dy_N_/Sm_N_, stemming from diagenetic alteration^39^ do not appear in our samples (Supplementary Fig. 7), suggesting that the calculated Ce anomalies were not significantly altered during diagenesis.

**Figure 8:**
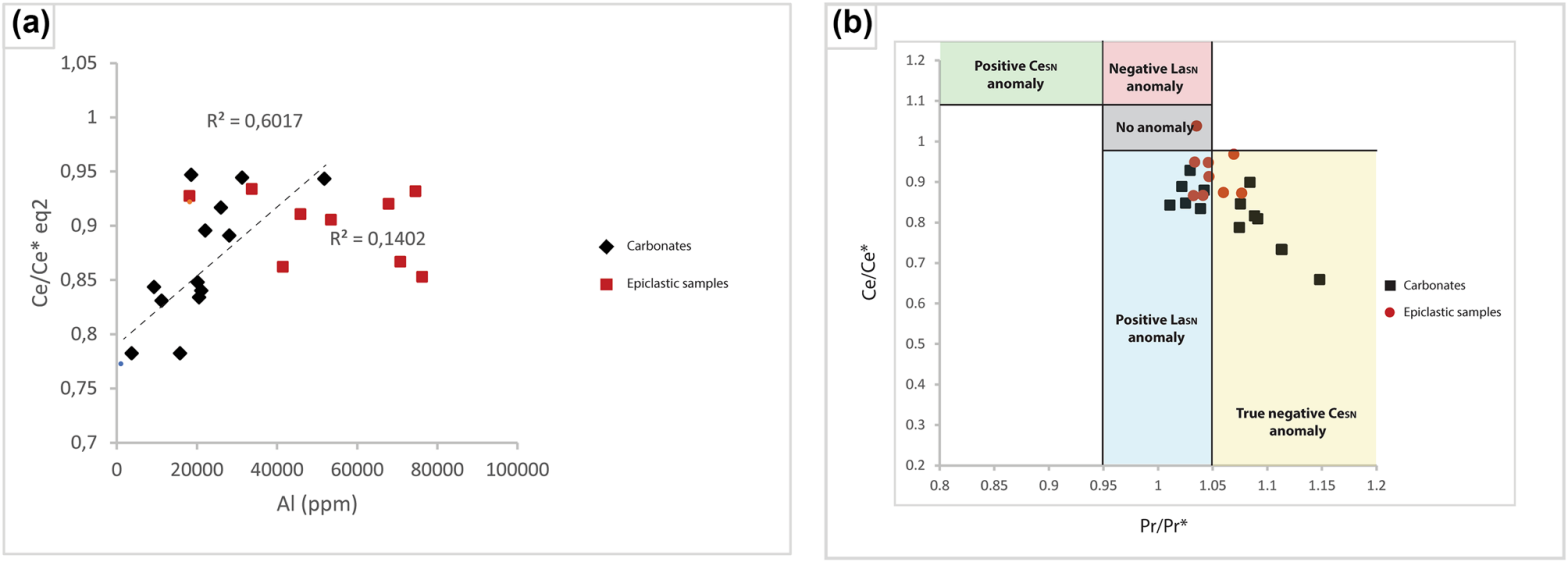
(**A**) Biplots of Ce/Ce* eq2 and Al showing a moderate positive correlation for carbonates and no correlation for epiclastic samples. (**B**) Amane Tazgart carbonates and epiclastic sediments in a graph of Ce/Ce* vs Pr/Pr* (Bau and Dulski^15^).

Detrital contamination is one of the most likely causes of deviations in carbonate REE distribution patterns from inherited primary features because of the relative enrichment of silicate derived REE compared to chemically derived carbonate REE content. Such effects could be ascertained by using concentrations and correlations among chemical elements such as bulk rock Al content and Ce/Ce* profiles of leachable carbonates^58,59^. The Ce/Ce* versus Al biplot demonstrates a moderate positive correlation for the carbonate samples (R^2^ = 0.6) and no correlation for the epiclastic samples (Fig. 8A). This means that the bulk Ce/Ce* data can be effectively used to infer the redox depositional conditions of the epiclastic samples but less so for the carbonates. Ce/Ce* was calculated and presented according to Bau & Dulski^14^ (Fig. 8B). As seen, the epiclastic samples cluster tightly in the negative Ce and positive La anomaly fields, with three samples showing deposition in oxic waters.

**Figure 9:**
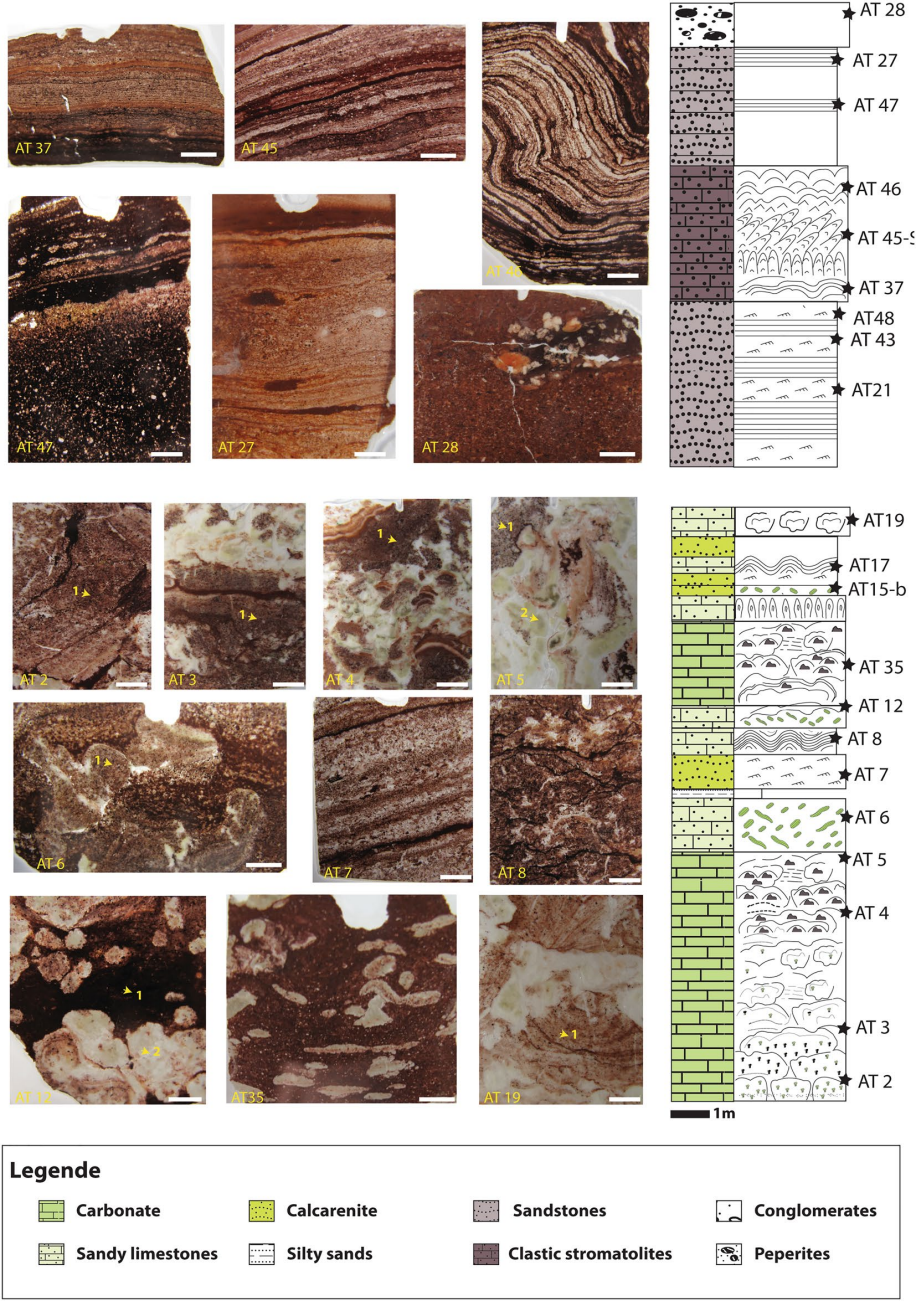
Detailed lithostratigraphic column associated with thin section pictures of the different analyzed samples of the Amane Tazgart succession (the lithostratigraphic column is modified from chraiki et al.^31^). Yellow arrows mark the analyzed parts of the samples (1 corresponds to primary fabrics, 2 correspond to early diagenetic phases).

Trace element concentrations for U, Th, Cu, Zn, Ni, Co, V, and Cr, as well as Ni/Co, V/V + Ni, V/Cr, Cu/Zn, and U/Th elemental ratios, commonly predict sediment redox depositional condition^27,28^. Pearson’s correlation analysis for the carbonates shows a strong positive correlation between V, Ni, and Al, a moderate positive correlation with Co, Cr, and U, and no correlation with Zn and Cu (Supplementary Table 8). These observations indicate that these elements are not authigenic or of dissolved water column origin, but due to detrital input, except for Zn and Cu. For the epiclastic samples, Pearson´s correlation analysis illustrates a positive correlation between Th, V, Co, and Al, and no correlation with the rest of the redox-sensitive elements (Supplementary Table 9), pointing to the authigenic or water column origin of Cr, Cu, Ni, U, and Zn in the epiclastic samples. Furthermore, and as previously stated, the Amane Tazgart samples are characterized by high EF values for Cu, Zn, and U (Supplementary Table 5), which means that the Cu/Zn ratio can be used as a redox proxy for both the epiclastic and carbonate deposits. The Cu/Zn ratio is proposed as a valid proxy for ancient redox conditions^26,60–62^. In reducing environments, the precipitation of Cu is favored over Zn, while they have about the same solubility in oxidizing environments. Thus, according to Hallberg^61^, high Cu/Zn ratios indicate reducing depositional conditions, while low ratios suggest highly oxidizing conditions. Except for AT3-C, AT19, and AT35, most of the carbonate samples display low Cu/Zn ratios, ranging from 0 to 0.82 (Fig. 6). These imply likely anoxic environments for AT3-C, AT19, and AT35 and highly oxidizing conditions for the rest of the carbonate samples. The low Cu/Zn ratio in the totality of the epiclastic samples, ranging from 0.05 to 0.64, which is taken to indicate highly oxidizing depositional environments, in full agreement with the Ce anomaly data. In facies formed under anoxic conditions, trace elements frequently show significant enrichment^63^. Mo EF, Cu EF, Zn EF, Cd EF, and As EF were used as redox proxies in the current study. As EF and Cd EF show the same patterns, expressing high enrichment in trace elements for carbonate samples and moderate enrichment in the epiclastic samples (Fig. 6). Samples with high Cu/Zn ratios are also highly enriched in the cited trace elements compared to the others, consistent with their anoxic depositional environment. The subsurface or pore water conditions were assessed from the early diagenetic phases of samples AT5-V and AT12-S as references (Fig. 9). These cements are highly enriched in redox-sensitive trace elements (Supplementary Table 5) and express high Cu/Zn ratios of 1.6 and 2.51, respectively, which may reflect anoxic pore water interface^61,64^. Thus, it appears that the carbonate lithologies formed in sub-oxic to oxic conditions and the epiclastic facies in fully oxygenated waters.

It is thought that As was a major component of Earth’s earliest oceans and that it played a crucial role in biological evolution^3,65–67^. In reduced environments, highly mobile As(III) predominates, conditions believed to have been prevalent in the anoxic Archean biosphere^67^, a time when As(III) detoxification is thought to have first evolved^68^. The oxidized As species, As(V), first rose to global prominence during the Paleoproterozoic Great Oxidation Event, (GOE) some 2.45 billion years ago^67^. Thus, the distribution and prevalence of As(III) and As(V) in the different lithologies have important implications for deducing the depositional environment, redox, and microbial activity.

In this regard, petrographic studies show that the microbialites preserve pristine carbonate phases and primary microbial fabrics^69^. However, they are slightly affected by early diagenesis, including dissolution and filling vugs with botryoidal and blocky/drusy mosaic calcite and late silicification^30^. The concentration of As in the studied microbialites presents a diffused pattern observed mainly within the mesoclots affiliated with the thrombolitic and composite microbialites, being concentrated within fine-grained clotted micrite (Fig. 2b). Whereas condensed As patterns are associated mostly with iron-rich stromatolitic microbial mats in the carbonates and epiclastic deposits (Fig. 2c–f). Sparry or botryoidal calcite in the same mapped samples does not concentrate As, which could be explained by the effect of early diagenesis that has likely affected the distribution of As in these diagenetic products, unlike the well preserved primary microbial features.

In line with previous observations above, As speciation in these different mat types is characterized by the co-occurrence of As(III) and As(V) in the calcite patterns (Fig. 3), and the condensed iron-rich As phases mainly in the As(V) oxidation state (Fig. 4). Similarly, microbialites of modern extreme volcanic crater lakes are marked by As(III) enrichment relative to As(V) in diffused calcite patterns, with the predominance of As(V) in globular As-rich phases located within organic matter^70^. Like the conditions identified in the present study, these crater lakes are characterized by elevated salinity, pH, and elevated As concentrations^71^. The increased mobility of As(III) in comparison to As(V) under alkaline circumstances was shown to account for this variation in the distribution of As compounds^72^.

According to laboratory studies, abiotic incorporation of As in calcite structures is mostly associated with As(V)^73^, while biogenic reactions incorporate As(III) and As(V)^74^. According to Bardelli et al.,^75^ bacteria may have been a significant factor in promoting As(III) uptake in the calcite lattice. The presence of As(III) and As(V), linked to the diffused As patterns in the crystalized biogenic calcite particles, strongly indicates that the incorporation of As into the fossilized carbonates was microbially mediated (Figs. 2b, 3). Typically, As(III) enters the cells through aquaporin membrane transporters^68,76,77^ or can be generated within the cytoplasm when As(V) is trafficked across the cell membrane through phosphate membrane transporters and then reduced to As(III) by As(V) reductases for detoxification^67,78^. Intracellular As can be further transformed into various methylated arsenicals and associated with biomass to further attenuate toxicity or as biological weapons^68,77^. In fact, because of its high toxicity, almost all known modern prokaryotes are involved in As detoxification of some form or kind^3,71^. These include specialized lineages involved in chemoautotrophic and photoautrophic As(III) oxidation, as well as respiratory As(V) reduction to As(III) species^71^.

Considering this view, the considerably enrichment of As, with an EF of up to 919, relative to UCC in the Ediacaran Amane Tazgart volcanic lake ecosystem can predictably be linked to their hydrothermal depositional environment, redox and biological activity. For example, such extreme As enrichments commonly characterize modern shallow submarine hydrothermal systems where hydrothermal fluids contain > 3000 times more As than seawater^79^ and alkaline volcanic lakes^80^. As a consequence, As detoxification is indispensable for microbial communities to succeed in these modern As-rich settings^3,71,78,81^. Because of the ancient origin of biological As detoxification^76^ and the abundance of similar As redox species in modern aquatic environments as was then, it is implied that comparable As detoxification mechanisms would have been required for the Amane Tazgart microbialites.

Further still, because As(V) is a strong analog of the phosphate macronutrient, its abundant accumulation in the water column would have posed a serious challenge to extant life, by for example competing with phosphate for uptake into cells through phosphate membrane transporters^78^. This interaction has been identified as a key cause of phosphate limitation in modern As(V)-rich alkaline volcanic lakes^78^ and in seawater settings^82^. Critically, phosphorus is crucial for all life, being the backbone of nucleic acids, and cell membranes, and serves as the cell’s main energy trafficking molecule^83^. In oxic bottom water environments, Fe (III) oxide-oxyhydroxides co-precipitate phosphate from the water column and sediment pore waters at the oxic/anoxic interface^84^. Below this zone in the anoxic sediments, the Fe (III) oxide-oxyhydroxides are dissolved by reductive processes, and dissolved P is returned to the overlying water column or sediment pore water.

Our study shows that P is absent or below detection in the carbonate samples, which if present should have been accumulated as abundant apatite carbonate minerals, but was instead co-enriched with Fe present principally as hematite^30,69^ in most of the epiclastic samples (Supplementary Table 1). A well-supported positive linear correlation between Fe_2_O_3_ and P_2_O_5_ in the epiclastic deposit (Supplementary Fig. 8), suggests P and As sequestration may be linked to Fe (III) oxide-hydroxide precipitation dynamics^84^, as a function of oxygen availability in the epiclastic stromatolites. The more variable redox conditions in the carbonates imply increased reductive recycling of Fe (III) oxide-oxyhydroxide bound-P and As was likely more common than in the strongly oxygenated epiclastic microbial mats. Consequently, stress resulting from phosphate scarcity would therefore have varied significantly during the deposition of the epiclastic and carbonate microbialites. Particularly, high-affinity phosphate membrane transporters would have been required to scavenge small amounts of available P when dissolved As(V) to phosphate ratios rose dramatically, similar to observations in comparable modern As-rich soda lakes^78^ and hydrothermal ecosystems^3^.

## Conclusions

Our high-resolution biogeochemical reconstruction demonstrates ecosystem succession by extremophilic Ediacaran microbial communities, driven by climate change, hydrothermal activity, and geochemistry. The underlying thrombolitic microbialites represent extremophiles that subsisted in oxygen-poor As-rich high-temperature hydrothermal saline to brackish waters at a time when the climate was cold/dry. The overlying epiclastic stromatolites evolved in a freshwater body in a warmer/wetter climate. They thrived in an oxygen-rich environment marked by lesser hydrothermal influence. This study provides a rare glimpse into how changing environmental chemistry structured adaptation and shifts in extreme microbial communities towards the end, and possibly, throughout much of Precambrian times.

## Methods

### Geological setting and sample preparation

The Amane Tazgart succession crops out ~ 25 km southward of Ouarzazate city, and in the southern part of the Ediacaran Saghro massif of the Anti-Atlas belt (Supplementary Fig. 9). It is a lens-shaped unit, less than 1 km wide and up to ~ 15 m thick. More details about the geological background of this location can be found in the supplementary file (Supplementary Note 3). To understand the diversity of the Amane Tazgart microbial deposits and the biogeochemical conditions in which they thrived, several primary fabrics from each facies were selected and analyzed from the carbonates (AT2 to AT19, Fig. 9) and epiclastic microbialites (AT37 to AT 28, Fig. 9). The early diagenetic phase was also selected from two samples of carbonate microbialites (AT5 and AT2, Fig. 9) to decipher the physical and chemical influence of pore water during burial diagenesis and also to compare with data from pristine carbonate. Whole rock geochemistry was performed on the epiclastic stromatolites and non-microbialitic samples because they are less affected by diagenesis.

### Whole-rock geochemistry

Geochemical analyses were carried out on 13 microbialites and 10 host sediments and analyzed at “Service d’Analyse des Roches et Minéraux” (SARM) of the “Centre de Recherches Pétrographiques et Géochimiques” (CRPG), Nancy, France. Based on a detailed petrographic study, 23 mineralogical phases were cautiously sampled from 21 polished slabs, using a 0.1 to 1 mm diameter microdrill under a stereoscopic microscope to avoid contamination. Each sample was powdered in an agate mortar, and approximately 0.5 g was fused with lithium metaborate (LiBO_2_) and dissolved in dilute nitric acid (HNO_3_). Major elements, trace, and rare earth elements data were obtained using inductively coupled plasma atomic emission spectrometry (ICP-AES) and inductively coupled plasma mass spectrometry (ICP-MS), respectively, following the techniques described in Carignan et al.^85^. Different statistical methods were applied to acquired data, including Pearson’s correlation coefficient and principal component analysis (PCA), using the XLSTAT software. Information about the REY data normalization, and the method of calculation of Ce anomalies, Pr anomalies, Eu anomalies, enrichment factor, chemical index of alteration, and statistical analysis can be found in the supplementary file (Supplementary Methods).

### Synchrotron radiation nano-XRF imaging

Elemental distribution maps were obtained by Synchrotron Radiation scanning X-ray fluorescence (SR-XRF) at the Nanoscopium hard X-ray nanoprobe beamline, Synchrotron Soleil, Paris, France^86^. Polished sample sections of 0.8–1.2 mm thickness were used for measurements. The monochromatic incident X-ray beam was focused on the sample position by using Kirckpatrick-Baez (KB) nano-focusing mirrors. For XRF imaging, the incident beam energy was tuned to 12.8 keV by the Si(111) double crystal monochromator, above the As K-edge but below the Pb-L3 edge, in order to obtain unambiguous As distribution maps. The correction of the reconstructed elemental distribution maps for XRF spectral overlap, dead-time correction, etc. were performed by Matlab codes developed by the Nanoscopium beamline.

### X-ray absorption near edge structure spectroscopy (nano-XANES)

Nano-XANES measurements were performed at the Nanoscopium beamline of Synchrotron Soleil. Two thin Si diodes were used to measure the I_0_ intensity in front of the KB and the I_Tr_ transmitted beam intensity behind the sample, respectively. In addition, a thin Au foil and a dedicated Si diode were inserted behind the I_tr_ monitor. In order to cover the As-K and Au-L_3_ absorption edges, the energy of the incident X-ray beam was scanned in the 11.825–11.935 keV energy range using 0.5 eV energy steps. This permitted energy calibration of each XANES spectrum compared to the inflection point of the simultaneously measured Au-L3 edge. The As XANES was measured on several characteristic points for samples AT2 and AT4 (Fig. 9).

## Supplementary Information

Supplementary Information.

## Data Availability

All data generated or analyzed during this study are included in this published article and supplementary information files.
