# Supplementary material for: Blooming of a microbial community in an Ediacaran extreme volcanic lake system
Source: Sci Rep. 2023 Jun 5;13:9080. doi: 10.1038/s41598-023-36031-5 (PMC10241817; doi:10.1038/s41598-023-36031-5)
Supplement: Supplementary file 1 — Supplementary Information. [file 41598_2023_36031_MOESM1_ESM.pdf]

Supplementary information for:

Blooming of a microbial community in an Ediacaran extreme volcanic lake system

Ibtissam Chraiki[1], Ernest Chi Fru[2], Andrea Somogyi[3], El Hafid Bouougri[1], Olabode Bankole[4], Mohamed Ghnahalla[4], Abderrazak El Albani[4*]

[1]Department of Geology, Faculty of Sciences-Semlalia, Cadi Ayyad University, Marrakesh, Morocco

[2]Centre for Geobiology and Geochemistry, College of Physical and Engineering Sciences, School of Earth and Environmental Sciences, Cardiff University, Cardiff, UK

[3] Synchrotron Soleil, Saint-Aubin, France

[4]CNRS IC2MP UMR 7285, University of Poitiers, Poitiers, France

Supplemental file contents:

Supplementary text

Supplementary Figures 1 to 9

Supplementary Tables 1 to 9

Supplementary captions

Supplementary References

Supplementary Note 1: Trace element systematics

For the 54 trace elements measured, high concentrations were recorded for As, Ba, Cr, Cu, Pb, Sb, U, V, and Zr. Arsenic content decreased upward in the section with a general variation from 108–277 ppm, averaging 189 ppm in the carbonates, 22.7–77.2 ppm with an average of 49.62 ppm in the epiclastic deposits (Supplementary Table 2). Average As concentrations in the carbonates are ~3.81 times over those measured in the overlying epiclastic facies and ~126.16 times in excess of upper continental crust (UCC) concentrations[1]. Ba is the most abundant trace element in all samples, averaging 100.89 ppm and 522.1 ppm in the carbonates and the epiclastic deposits, respectively (Supplementary Table 2). Overall, the samples are enriched in Pb compared to the UCC, with an average of 3.92 ppm and 10.12 ppm in the carbonates and epiclastic samples and exceeding the UCC concentration by 3.92 and 10.12 times, respectively. Average carbonate Cu concentrations of 38.34 ppm exceed UCC composition by 1.5 times, whereas an average of 11.82 ppm in the epiclastic deposit show deficiency compared to the UCC (a mean of 25 ppm). The carbonate Sb values show a slightly varying pattern relative to the epiclastic deposit. They exhibit a mean of 4.4 ppm in the carbonates, which tended to fluctuate from 0.42–8.23 ppm, being 22 times above UCC content. The average of 7.74 ppm, spread between 2.69-14 ppm in the epiclastic material, showed 38.69 times excess of UCC concentrations. U composition expresses values 2.71 and 3.9 times above the geochemical background of the UCC[1] in carbonates and epiclastic samples, respectively. On average, V was shown to have elevated concentrations up to 14.38 and 39.92 times over UCC concentrations for the carbonates and the epiclastic samples, respectively. Mo remained below the detection limit of 0.5 ppm in most of the carbonates and epiclastic samples, except AT2, AT3-C, AT12-S, and AT27, which had values ranging from 0.63–15.1 ppm (Supplementary Table 2).

Supplementary Note 2: Rare earth elements (REE) and Yttrium (Y) systematics

The carbonate microbialites show general REY patterns characterized by depletion in light rare earth elements (LREE), enrichment of middle rare earth elements (MREE), and a variation from relatively flat to being slightly depleted in heavy rare earth elements (HREE) (Figure 1a). Relative to PAAS, the epiclastic samples display slight light LREE depletion and MREE enrichment, while a slight enrichment in HREE was detected for two samples (Figure 1b). The others showed flat to relatively depleted patterns (Figure 1b). A slight enrichment in light rare earth elements (LREE) compared to (HREE) was observed in AT3-C, AT5-V, AT12-S, AT48, AT46, and AT47 with Pr_N/Yb_N= 1.11, 1.26, 1.16, 1.23, 1.72, and 1.28, respectively (Supplementary Figure 5, Supplementary Table 3).

Supplementary Note 3: Geological setting

The Moroccan Anti-Atlas is an ENE–WSW oriented belt bounded northward by the Variscan South-Atlas strike-slip Fault (SAF), and forms the foreland of the Variscan belt which expose a Proterozoic basement and an Upper Ediacaran-Paleozoic slightly folded cover [2–4] (Supplementary Figure 8a). In the Anti-Atlas inliers, the ca. 2 Ga Eburnean Paleoproterozoic to ca. 890-580 Ma Pan-African-Cadomian Neoproterozoic basement is unconformably overlain by the ca. 577-547 Ma Upper Ediacaran Ouarzazate Group, which forms a thick and regionally extensive volcano-sedimentary wedge associated with intrusive plutonic suites [5–9]. In the Saghro massif (Supplementary Figure 8a), the Upper Ediacaran volcanic and volcaniclastic Ouarzazate Group and their coeval subvolcanic intrusive rocks are subdivided into the lower

Mançour Group and the upper Imlas Group (labeled respectively as XIIIm and XIIIs in the geological map of Tiwit[10] . They are separated by an angular unconformity related to the tilting of block fault, and are crosscut by the plutonic Tanghourt suite[9,11–13].

Most of the volcanic rocks are peraluminous to metaluminous and range from basalt, andesite to rhyolite, with high-K calc-alkaline to shoshonitic affinities [9]. Their structural, sedimentary, and volcanic features indicate a wide caldera containing several subaerial volcanic centers with effusive activity and subaerial clastic sedimentation. The main known Caldera complexes in the central and eastern Anti-Atlas are Achkoukchi in Sirwa, Oued Dar'a, and Qal'at Mgouna in Jbel Saghro[8,9,13]. In the southeast part of the Saghro massif (Supplementary Figure 8a-b), the Oued Dar'a caldera forms in a pull-apart graben, and consists of a wide rectangular volcanic structure, bounded by two NE-SW strike-slip faults[14]. This tectonic and volcanic trough is mainly filled with the volcanic and volcaniclastic rocks of the lower Ouarzazate Group, which are crosscut by rhyolitic dykes. The dominating facies are ash-flow tuff of dacite and rhyolite composition.

The tuff from the Oued Dar'a caldera yielded a SHRIMPH U-Pb zircon age of 574 ± 7 Ma, which is approximately similar to the age of 571 ± 7 Ma obtained from a tuff in the northern margin and interpreted as the age of the Oued Dar'a Caldera-fill and as the time of the last major eruption from this Caldera (Supplementary Figure 8b)[9]. The northeast margin of Oued Dar'a Caldera is crosscut by pink granite, whereas the southwest margin forms a classical sequence of caldera collapse margin, dominated by volcaniclastic deposits and in which thin and minor lacustrine beds are preserved[2,9,15,16]. Among these sparse meter-thick lacustrine carbonate-bearing layers is the famous succession of Amane Tazgart (Amane-n'Tourhart sensu Choubert & Faure Muret,[16]), labeled in the 1/50.000 geological map of Tizgui as NP3cs[17]. This locality crops out along the national road of Ouarzazate-Agdz, ~25 km south of Ouarzazate city.

The Amane Tazgart succession forms a lens-shaped unit, less than 1 km wide and up to ~15 m thick in the central part. The succession is gently folded, slightly dipping toward the WNW, and divided by an NNE-SSW fault into two compartments[18]. It is underlain by grey aphanitic andesite flows and overlain by a volcano-sedimentary succession including andesitic lava flows, peperites, and epiclastic micro-conglomerate and sandstone rich in volcanic clasts. All these rocks including the Amane Tazgart are crosscut by doleritic dykes up to 4 m thick. The surrounding depositional setting dominated by the subaerial volcanic and sedimentary setting of the Oued Dar'a Caldera, as well as the overall facies features of the Amane Tazgart suggest a short quiescence episode of volcanism and lacustrine deposits in ephemeral ponds [2,9,15,16,18,19].

The Amane Tazgart succession can be divided broadly into a carbonate dominated lower part and an epiclastic-dominated upper part (Figure 10). In both parts, a variety of distinct microbial deposits and related fabrics, are interbedded with non-microbial deposits[18]. Microbial deposits show diversity in shapes and fabrics and preserve evidence for primary microbial accretion structures and early diagenetic processes. The lower part consists mainly of thrombolites, and composite microbialites usually with irregular to patchy mesoclots, or occasionally arranged as a dendroidal pattern[18]. The fabrics contain peloidal clots surrounded by primary calcite cement. Tow beds of flat large domal stromatolites up to 30 cm thick are intercalated within thrombolitic and composite microbialites (Figure 10). They are characterized by an alternation of tufted microbial mats, micritic laminae, and microsparitic laminae[18]. The upper part comprises of a

meter-thick stromatolite bed, arranged into several growth forms. This bed starts with flat to wavy laminated stromatolites that grades to small-scale linked stromatolitic columns. They are overlain by decimeter to meter thick domal stromatolites with low convex laminae. The overall changes in shape appear controlled by water depth and physical environmental conditions[18]. Microbial fabrics in the thrombolites and stromatolites were partially disrupted and vanished during diagenesis and hydrothermal fluid seepage. The resulting fabrics record a complex diagenetic story. Primary calcite cement geopetally filled the internal porosity, whereas both microbial fabrics and cement generations were silicified during the late diagenetic stage[18,19]. Previous works on the Amane Tazgart deposits and their equivalents in the Anti-Atlas described the general trend of these deposits, facies diversity, and diagenetic processes with various stages of cementation and silicification, linked to hydrothermal fluids[15,16,18,19]. However, the geochemistry and the environmental parameters controlling the development and growth of these extremophile microbial features remain to be fully described, which is the aim of the current work.

Supplementary Methods

1.1 Whole-rock geochemistry

REY data were normalized to Post Archean Average Shale (PAAS, subscript SN, Taylor and MacLennan[20]), calculated as $[REE_X]/[REE_X{*}]$, where $[REE_X]$ is the measured concentration of rare earth element X, and $[REE_X{*}]$ is the concentration of element X predicted by extrapolation or interpolation from adjacent PAAS-REEs.

Ce anomalies (Ce/Ce*) were calculated using two equations:

Eq (1) $$\frac{Ce_{SN}}{Ce^{*}_{SN}} = \frac{2*[Ce]_{SN}}{[La]_{SN}+[Pr]_{SN}}$$ [21]

Eq (2) $$\frac{Ce_{SN}}{Ce^{*}_{SN}} = \frac{[Ce]_{SN}}{([Pr]_{SN})^2/\,[Nd]_{SN}}$$ [22]

Praesodymium anomalies (Pr/Pr*) were calculated using the equation of Bau and Dulski (1996):

$$\frac{Pr_{SN}}{Pr^{*}_{SN}} = \frac{2Pr_{SN}}{Ce_{SN}+Nd_{SN}}$$

Europium anomaly was calculated using the equation:

$$Eu/Eu*= \frac{2*[Eu]_{SN}}{[Sm]_{SN}+[Gd]_{SN}}$$

The enrichment factor (EF; cf. Tribovillard et al[23].,) with respect to the average shale was employed to evaluate whether an element was authigenically enriched or depleted. $X\text{-}_{EF}$ (enrichment factor of element X) was calculated by the following equation: $X\text{-}_{EF} = (X/Al)_{sample}/(X/Al)_{ref(PAAS)}$[23,24].

1.2 Chemical index of alteration (CIA)

CIA is calculated as: $CIA=100\times molar[Al_2O_3/(Al_2O_3+CaO^*+Na_2O+K_2O)]$. CaO* corresponds only to CaO in the silicate fraction[25]. Our correction procedure for CaO* follows that in McLennan (1993): (1) CaO is first corrected for that residing in apatite using P_2O_5 data (CaO′

= $CaO - 10/3 \times P_2O_5$), and (2) if CaO′ is superior to Na_2O, the final CaO* value is set equal to Na_2O, however assuming CaO′ is under Na_2O, the final CaO* is set equivalent to CaO′. The correction formula for K metasomatism followed that of Panahi et al.,[26]:

$m = K_2O/(Al_2O_3 + CaO^* + Na_2O + K_2O)$ for the protolith sample

K_2O_{corr} = molar $[m\times Al_2O_3 + m\times (CaO^* + Na_2O)]/(1 - m)$

CIA_{corr} = molar $Al_2O_3/(Al_2O_3 + CaO^* + Na_2O + K_2O_{corr})$

1.3 Statistical analysis

In this study, we used different methods including Pearson's correlation coefficient and principal component analysis (PCA). Pearson's correlation coefficient is a statistical measure of the intensity of a linear relationship between paired data. The correlation coefficient always has a value from -1 to +1, with -1 indicative of an ideal negative correlation, +1 infers a perfect positive correlation, and a value of 0 implies that there is no linear dependency between the variables 0 indicating no correlation at all. The Pearson´s coefficient and (PCA) were carried out using XLSTAT software.

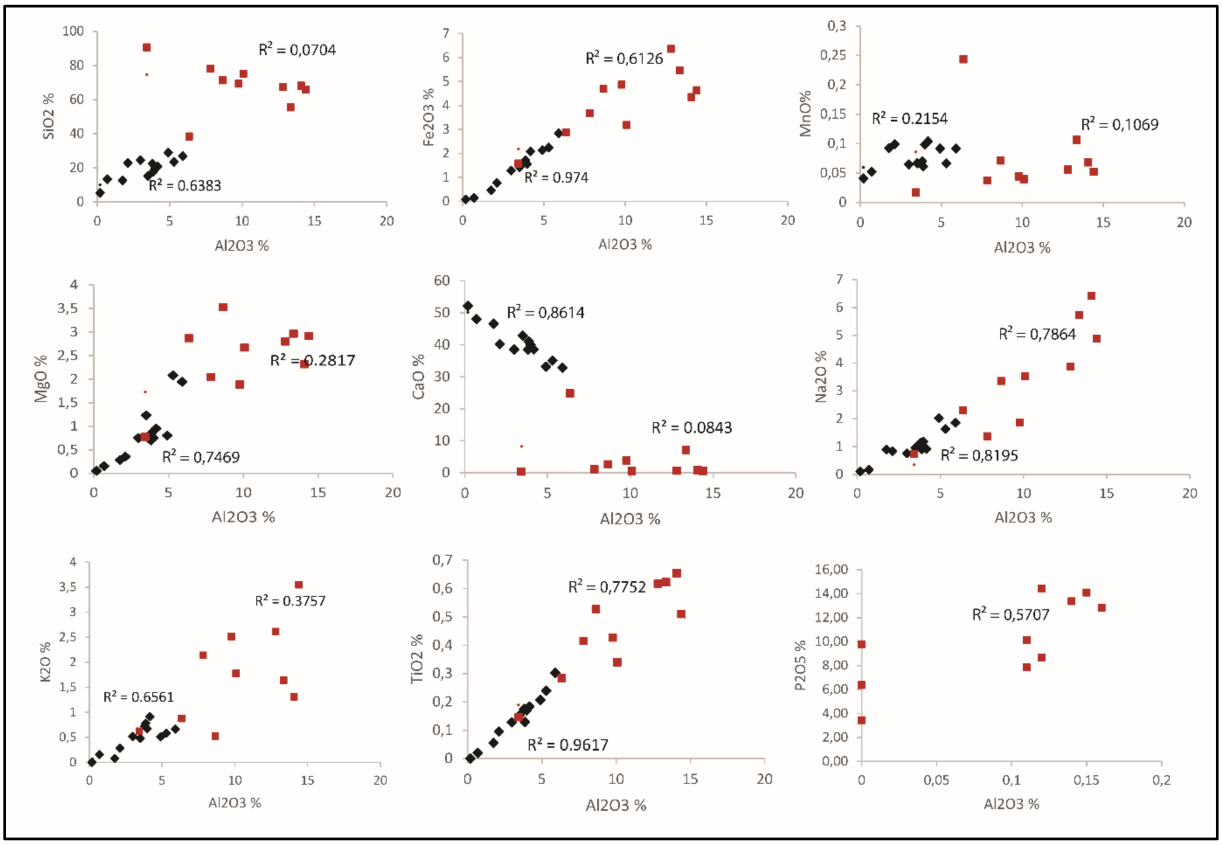

Supplementary Figure 1 Plots of major elements vs Al2O3 %

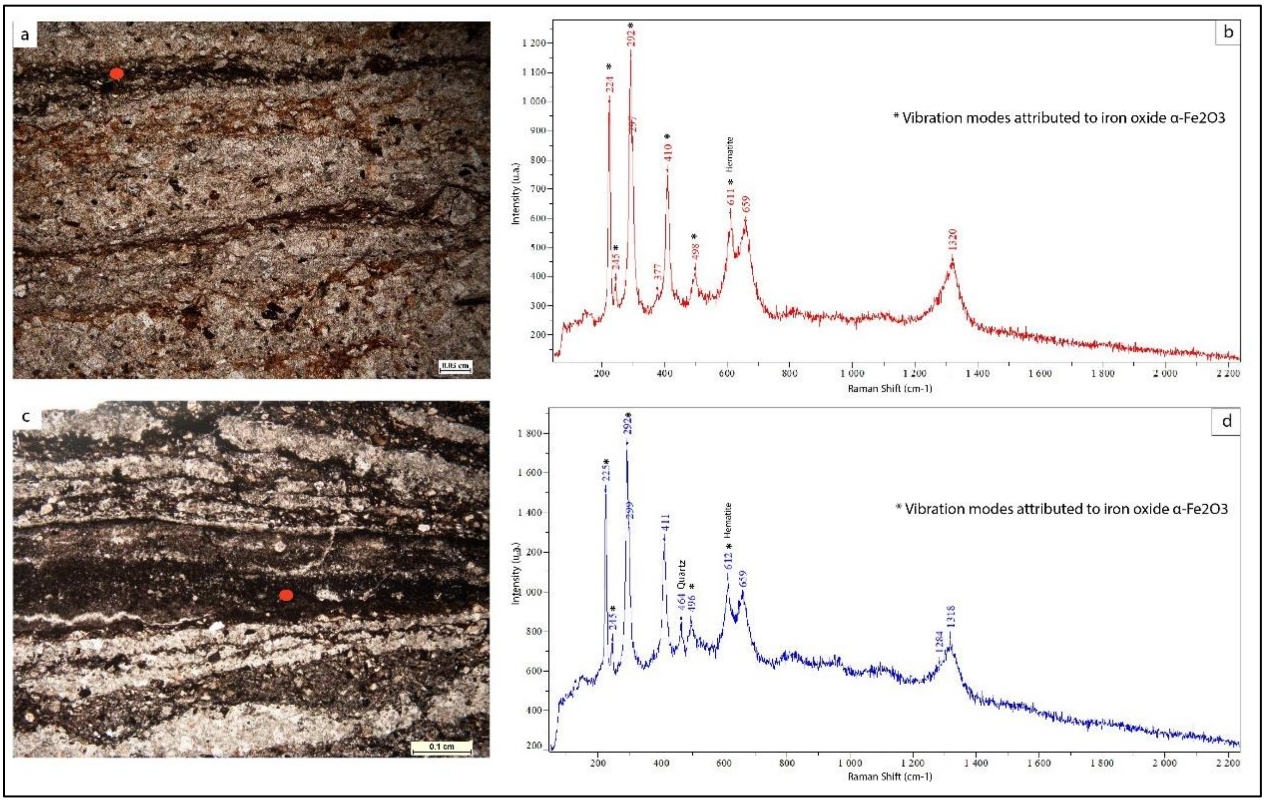

Supplementary Figure 2 a Thin section photomicrograph of a stromatolite composed of the alternation of iron crust and grain-sized laminae. b Raman spectra of the marked red point in (a). c Thin section photomicrograph of a stromatolite composed of iron laminae, sparitic crusts, and grain-sized microbial laminae. d Raman spectra of the marked red point in (c).

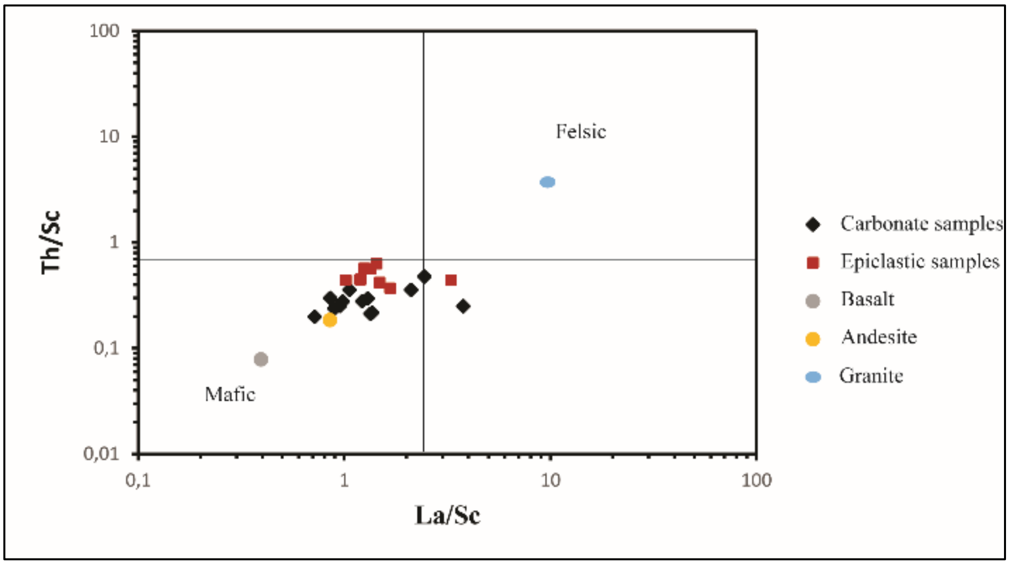

Supplementary Figure 3 Th/Sc versus La/Sc diagram demonstrating a mafic provenance of Amane Tazgart samples. Note: Andesite, basalt amd granite values are from Condie[27].

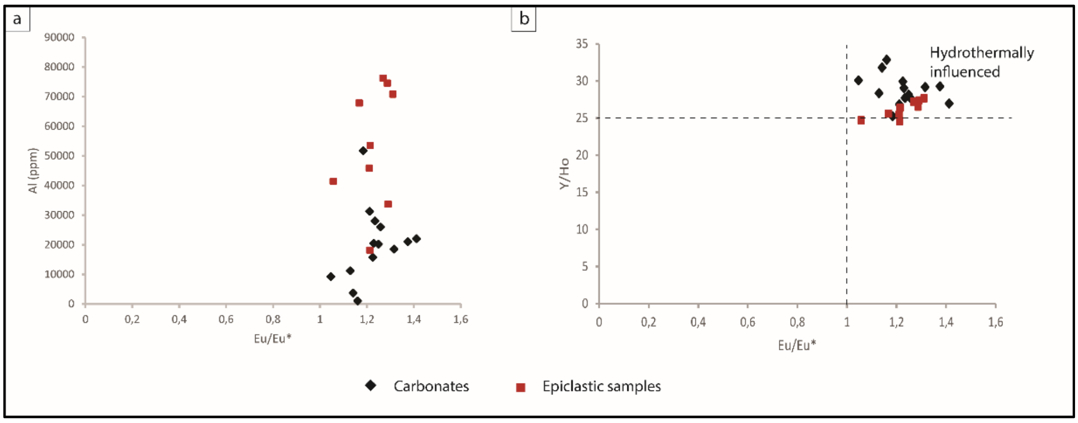

Supplementary Figure 4 a Al versus Eu/Eu showing no correlation. b Y/Ho versus Eu/Eu*[28].*

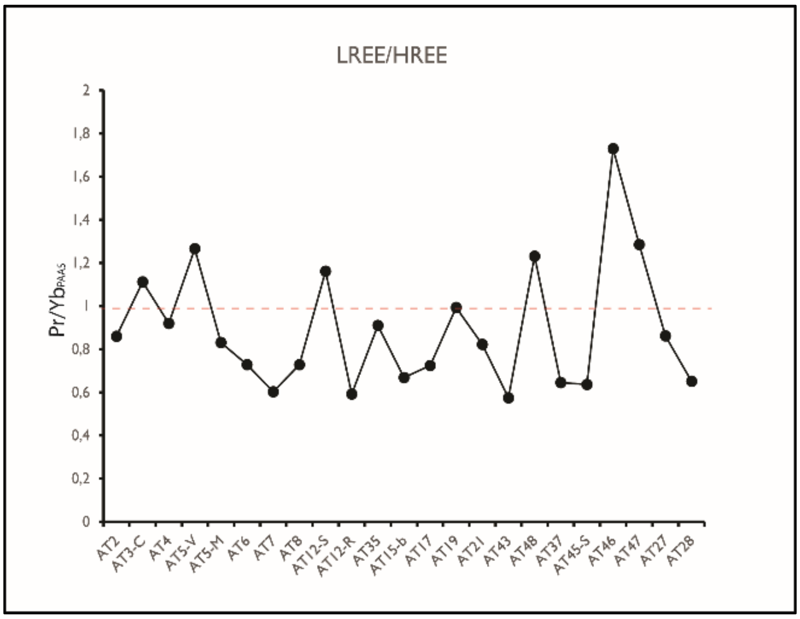

Supplementary Figure 5 Pr/Yb_{PAAS} diagram discriminating between samples enriched with LREE ($Pr/Yb_{PAAS}>1$) and those enriched with HREE ($Pr/Yb_{PAAS}<1$).

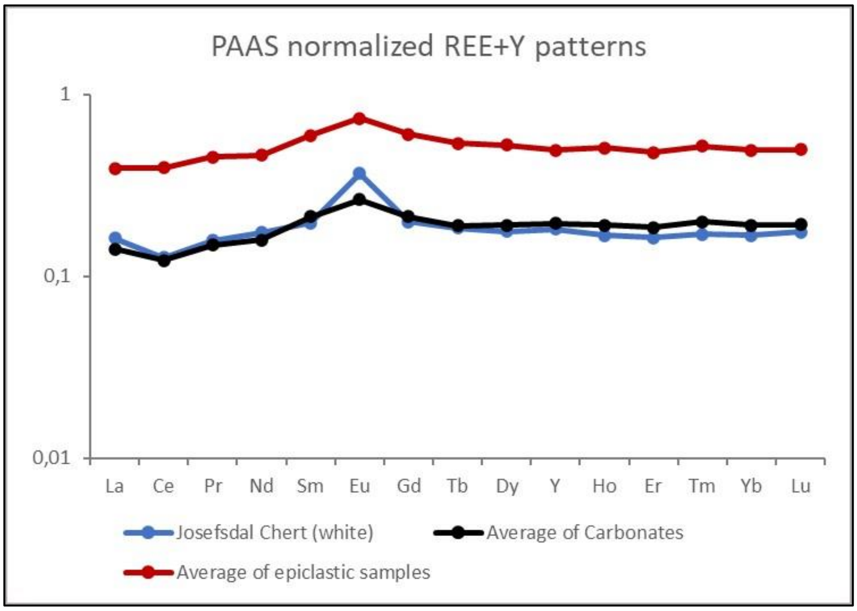

Supplementary Figure 6 PAAS normalized REE+Y spectra of the averages of carbonates and epiclastic samples, compared with REE+Y spectra of Josefsdal chert.

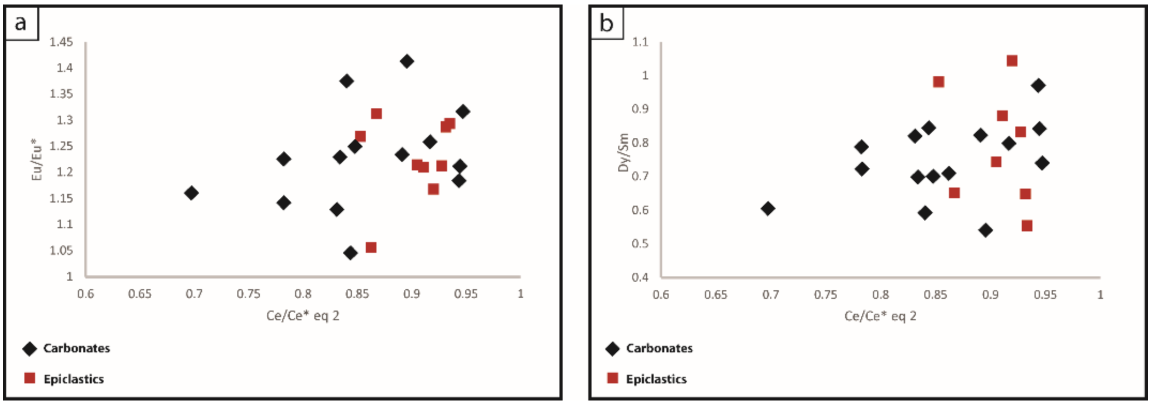

Supplementary Figure 7 Figure IV. 9 (a) Plots of Ce/Ce versus Eu/Eu* (Shields and Stille [29])*

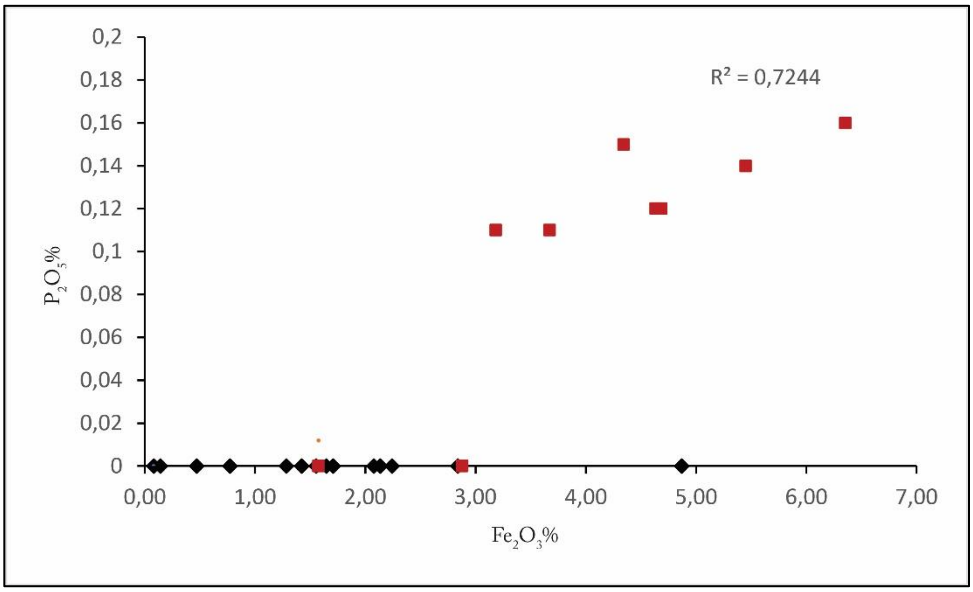

Supplementary Figure 8 Biplots of Fe_2O_3% and P_2O_5% showing no correlation for carbonate samples and a positive correlation for epiclastic samples.

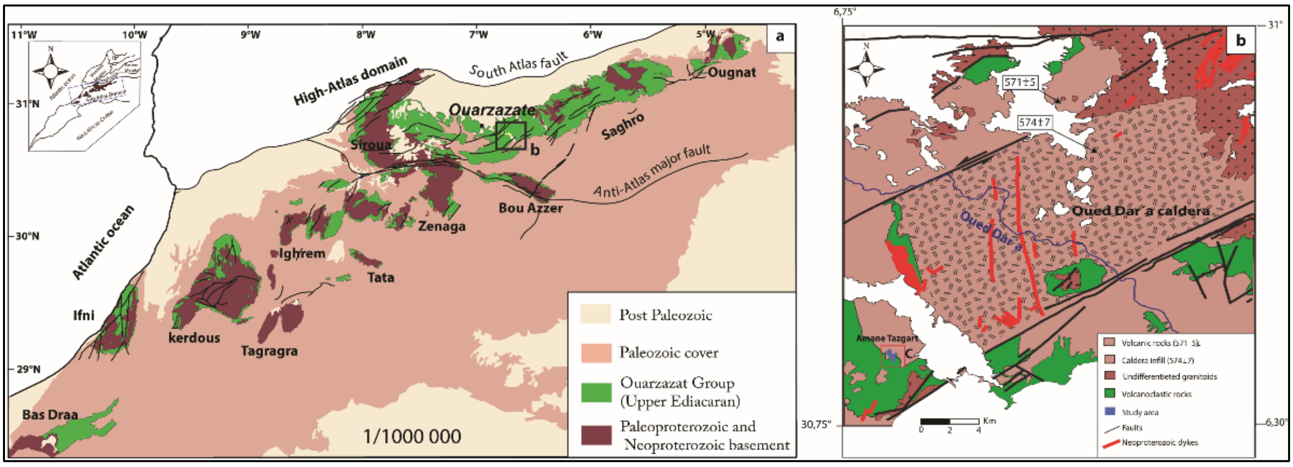

Supplementary Figure 9 a Simplified geological map of the Anti-atlas of Morocco. b Structural Map of Oued Dar'a caldera showing the localization of the studied area (modified from Chraiki et al.,[18]).

Supplementary Table 1 Major element data

Samples	SiO2	Al2O3	Fe2O3	MnO	MgO	CaO	Na2O	K2O	TiO2	P2O5
	%	%	%	%	%	%	%	%	%	%
AT2	19.48	3.98	1.55	0.098	0.75	39.98	1.17	0.67	0.17	< L.D.
AT3-C	20.76	4.17	2.08	0.10	0.95	38.54	0.91	0.92	0.18	< L.D.
AT4	17.65	3.87	1.71	0.061	0.86	41.07	0.90	0.78	0.13	< L.D.
AT5-V	5.20	0.19	0.08	0.041	0.05	52.13	0.10	< L.D.	< L.D.	< L.D.
AT5-M	22.43	3.82	1.65	0.070	0.70	38.50	1.13	0.72	0.18	< L.D.
AT6	15.19	3.50	1.42	0.067	1.23	42.85	0.95	0.48	0.15	< L.D.
AT7	26.76	5.91	2.84	0.092	1.94	32.80	1.85	0.67	0.30	< L.D.
AT8	24.47	2.98	1.28	0.064	0.75	38.55	0.76	0.52	0.13	< L.D.
AT12-S	13.29	0.70	0.14	0.052	0.15	48.00	0.16	0.16	0.020	< L.D.
AT12-R	69.21	9.78	4.87	0.044	1.88	3.75	1.86	2.51	0.43	< L.D.
AT35	12.66	1.75	0.47	0.092	0.28	46.56	0.89	0.08	0.055	< L.D.
AT15-b	23.43	5.30	2.24	0.066	2.08	35.09	1.63	0.58	0.24	< L.D.
AT17	28.90	4.91	2.14	0.092	0.80	33.16	2.02	0.51	0.21	< L.D.
AT19	22.93	2.11	0.77	0.099	0.36	40.21	0.84	0.28	0.095	< L.D.
AT21	77.96	7.83	3.67	0.037	2.05	1.09	1.36	2.14	0.42	0.11
AT43	67.24	12.82	6.35	0.056	2.80	0.64	3.88	2.61	0.62	0.16
AT48	68.05	14.08	4.34	0.068	2.31	0.78	6.42	1.31	0.65	0.15
AT37	90.51	3.43	1.58	0.017	0.77	0.33	0.73	0.62	0.15	< L.D.
AT45-S	71.23	8.66	4.68	0.071	3.53	2.64	3.35	0.53	0.53	0.12
AT46	38.26	6.36	2.88	0.24	2.87	24.80	2.31	0.88	0.28	< L.D.
AT47	55.41	13.37	5.45	0.11	2.96	7.04	5.72	1.64	0.62	0.14

AT27	75.13	10.10	3.19	0.040	2.67	0.48	3.51	1.78	0.34	0.11
AT28	65.71	14.41	4.64	0.052	2.92	0.58	4.87	3.55	0.51	0.12
PAAS	62.8	18.9	7.22		2.2	1.3	1.2	3.7	1	0.16
Average of carbonate	19.47	3.32	1.41	0.08	0.84	40.57	1.02	0.49	0.15	0.00
Average of epiclastic samples	67.87	10.08	4.16	0.07	2.47	4.21	3.40	1.75	0.45	0.09

187 *Supplementary Table 2 Trace elements Data*

Samples	As µg/g	Ba µg/g	Be µg/g	Bi µg/g	Cd µg/g	Co µg/g	Cr µg/g	Cs µg/g	Cu µg/g	Ga µg/g	Ge µg/g	Hf µg/g	In µg/g	Mo µg/g	Nb µg/g
AT2	154	163	0.58	0.1	1.52	4.17	20.7	6.5	15.8	4.67	0.57	0.83	< L.D.	1.08	1.37
AT3-C	259	222	0.63	0.15	1.13	6.76	79.3	8.38	179	5.53	0.65	0.96	< L.D.	15.1	1.43
AT4	166	354	0.55	0.09	1.37	5.18	18	4.81	11.4	4.97	0.5	0.83	< L.D.	< L.D.	1.36
AT5-V	271	16.1	0.1	< L.D.	1.34	0.15	2.4	0.21	30.6	0.2	0.08	0.09	< L.D.	< L.D.	0.06
AT5-M	251	54.9	0.46	0.06	0.92	3.4	15.8	4.47	14.3	4.63	0.6	0.91	< L.D.	< L.D.	1.39
AT6	205	92.1	0.48	0.09	1.1	6.34	20.9	5.41	14.6	3.88	0.47	0.81	< L.D.	< L.D.	1.33
AT7	123	85.5	0.63	0.43	0.81	9.55	49.4	6.09	5.6	6.21	0.83	1.84	< L.D.	< L.D.	2.69
AT8	108	47.4	0.44	0.08	1.3	3.48	30.4	6.3	< L.D.	3.6	0.64	0.79	< L.D.	< L.D.	1.32
AT12-S	183	59.4	0.11	< L.D.	1.35	0.62	5.5	1.32	23.6	0.9	0.23	0.14	< L.D.	0.63	0.28
AT12-R	48.9	270	1.37	0.25	0.1	8.98	65.2	23.1	14.2	12	2.06	2.93	0.04	< L.D.	4.69
AT35	134	27.4	0.14	0.11	1.36	2.17	10.9	0.47	124	1.79	0.25	0.53	< L.D.	1.21	0.76
AT15-b	136	88.8	0.56	0.12	1.06	8.98	31.4	5.12	15.8	5.25	0.65	1.15	< L.D.	< L.D.	1.88
AT17	277	48.5	0.4	0.15	0.76	4.45	37.1	5.63	2.8	4.27	0.75	1.18	< L.D.	< L.D.	2.03
AT19	191	53	0.16	0.07	0.86	3.67	14.5	1.85	22.7	1.99	0.42	0.68	< L.D.	< L.D.	1.18
AT21	48.7	296	1.04	0.23	0.08	11.7	43.8	25.6	6	10.7	2.94	2.89	0.04	< L.D.	5.01
AT43	30.1	679	2.14	0.28	0.05	12.2	65.9	21	30.1	19.1	2.7	4.58	0.08	< L.D.	7.77
AT48	36.5	511	0.97	0.15	0.06	8.91	96.6	1.68	5.1	13.2	1.64	4.35	0.04	< L.D.	6.82
AT37	22.7	189	0.52	0.11	0.03	3.92	21.5	7.26	4.8	4.9	1.71	1.69	< L.D.	< L.D.	1.93
AT45-S	77.2	113	0.82	0.2	0.06	10.8	72.8	1.71	4	11	1.57	3.58	0.03	< L.D.	5.99
AT46	46.6	181	0.58	0.13	0.07	8.16	39.3	0.8	31.4	8.39	1.25	1.82	0.06	< L.D.	3.29
AT47	42.3	705	0.99	0.25	0.1	10.9	85.5	1.51	4.8	14.2	1.64	3.68	0.05	< L.D.	5.83
AT27	66.7	821	0.79	0.23	0.03	10.6	38.7	4.79	9.4	10.6	1.43	2.77	0.03	7.23	4.98
AT28	76.5	1456	0.88	0.25	0.03	12	38.2	1.42	8.3	12.3	1.5	4.22	0.03	< L.D.	6.18
PAAS		650				23	110					5		1	19
Aver. of Ca	189.24	100.89	0.4	0.13	1.14	4.53	25.86	4.35	38.34	3.68	0.51	0.83	0	4.49	1.31

Aver. of Ep	49.62	522.18	1.01	0.21	0.06	9.81	56.75	8.88	11.82	11.64	1.84	3.25	0.05	7.23	5.25
UCC	1.5	550			0.098	17	83		25			5.8		1.5	12
Ca/Ep	3.82	0.19	0.40	0.63	19.83	0.46	0.46	0.49	3.24	0.32	0.28	0.25	0.00	0.62	0.25
Ca/UCC	126.16	0.18	0.00	0.00	11.67	0.27	0.31	0.00	1.53	0.00	0.00	0.14	0.00	2.99	0.11
Ep/UCC	33.08	0.95	0.00	0.00	0.59	0.58	0.68	0.00	0.47	0.00	0.00	0.56	0.00	4.82	0.44

Samples	Ni μg/g	Pb μg/g	Rb μg/g	Sb μg/g	Sc μg/g	Sn μg/g	Sr μg/g	Ta μg/g	Th μg/g	U μg/g	V μg/g	W μg/g	Zn μg/g	Zr μg/g
AT2	6.1	3.61	44.2	4.01	4.96	0.34	190	0.12	1.05	6.37	18.8	1.64	27.3	29.6
AT3-C	9.6	10.9	54.5	8.23	5.19	2.17	229	0.13	1.13	8.69	33.2	18.8	116	35.5
AT4	7.2	4.10	39.4	8.67	4.63	0.30	198	0.13	1.37	8.97	20.8	0.86	20.7	28.2
AT5-V	< L.D.	1.59	1.95	0.42	< L.D.	0.64	213	< L.D.	0.05	4.77	2.8	< L.D.	19.0	6.68
AT5-M	4.8	2.84	60.7	5.49	4.49	< L.D.	259	0.13	1.13	7.60	44.0	< L.D.	17.3	32.2
AT6	7.2	3.31	29.1	3.78	4.10	< L.D.	199	0.12	0.97	6.65	21.5	< L.D.	26.7	29.4
AT7	17.3	6.65	38.6	6.63	7.90	1.07	138	0.25	2.36	16.2	51.1	1.09	36.1	64.8
AT8	6.3	3.19	34.1	3.67	3.87	< L.D.	151	0.12	1.08	9.33	19.7	< L.D.	18.6	27.8
AT12-S	< L.D.	1.21	8.24	1.30	0.85	< L.D.	224	0.03	0.21	6.69	4.9	0.85	9.4	5.12
AT12-R	17.0	8.58	131	11.3	12.21	1.50	54.1	0.45	4.37	18.5	65.1	0.83	30.5	101
AT35	2.6	3.45	3.34	1.43	1.77	< L.D.	186	0.10	0.63	2.71	11.1	< L.D.	14.2	19.1
AT15-b	9.9	3.44	34.1	3.50	6.72	0.37	189	0.17	1.34	6.92	31.7	< L.D.	38.6	40.1
AT17	8.8	4.67	30.5	6.72	5.68	0.41	184	0.18	1.57	9.56	47.3	0.82	16.0	40.6
AT19	7.3	1.95	16.7	3.36	1.99	< L.D.	191	0.12	0.95	4.34	11.1	< L.D.	11.6	23.8
AT21	16.6	6.00	140	14.2	8.72	0.97	53.9	0.45	3.90	7.75	55.5	< L.D.	38.3	98.8
AT43	32.0	15.6	120	6.81	16.59	2.25	200	0.75	7.34	9.47	103	< L.D.	124	151
AT48	15.9	9.08	34.4	2.69	14.40	1.13	310	0.65	6.05	5.19	63.7	< L.D.	62.1	144
AT37	7.3	5.06	39.8	9.99	3.10	0.39	34.1	0.19	1.77	7.56	27.7	< L.D.	42.3	63.8
AT45-S	13.8	9.51	13.4	8.35	9.14	1.21	42.7	0.56	5.18	33.0	48.7	< L.D.	75.4	127
AT46	13.3	6.49	13.7	5.58	6.18	0.64	114	0.31	2.73	14.2	35.7	< L.D.	48.4	65.2

AT47	19.2	15.30	37.60	3.56	14	1.16	328.00	0.6	5.1	6.98	72.6	0.8	90.4	124.0
AT27	14.2	15.3	51.8	9.39	8.62	1.00	136	0.47	5.45	3.37	113	1.28	62.5	96.2
AT28	13.8	10.3	78.2	5.56	11.64	1.06	238	0.64	6.60	3.47	93.3	0.86	64.6	141
PAAS	55	20	160	200	16		200	1.2	14.6	3.1	150		85	210
Aver. of Ca	7.92	3.92	30.43	4.40	4.35	0.76	196.30	0.13	1.07	7.60	24.45	4.01	28.59	29.45
Aver.of Ep	16.30	10.12	66.04	7.74	10.44	1.13	151.02	0.50	4.85	10.95	67.87	0.95	63.88	111.18
UCC	44	0.5	112	0.2			350	1	10.7	2.8	1.7	2	71	190
Ca/Ep	0.49	0.39	0.46	0.57	0.42	0.67	1.30	0.26	0.22	0.69	0.36	4.24	0.45	0.26
Ca/UCC	0.18	7.84	0.27	22.00	0.00	0.00	0.56	0.13	0.10	2.71	14.38	2.01	0.40	0.15
Ep/UCC	0.37	20.25	0.59	38.70	0.00	0.00	0.43	0.50	0.45	3.91	39.92	0.47	0.90	0.59

*Supplementary Table 3 Data of Cerium anomalies (Ce/Ce*and Ce/Ce* Eq 2) calculated using the equation of Bau and Dulski[21] and Lawrence et al., [30], respectively, Praseodymium anomalies (Pr/Pr*) calculated using the equation of Bau and Dulsk[21], Europium anomalies (Eu/Eu*), Y/Ho and Pr/Yb ratios. Note : REY data were normalized to Post Archean Average Shale (PAAS).*

**Samples**	**Ce_N/Ce_N^***	**Pr_N/Pr_N^***	**Ce_N/Ce_N^* eq 2**	**Y/Ho**	**Eu_N/Eu_N^***	**Pr_N/Yb_N**
AT2	0.79	1.07	0.84	29.26	1.38	0.86
AT3-C	0.88	1.04	0.90	26.97	1.41	1.11
AT4	0.82	1.09	0.83	29.04	1.23	0.92
AT5-V	0.66	1.15	0.70	32.85	1.16	1.27
AT5-M	0.85	1.08	0.85	28.16	1.25	0.83
AT6	0.84	1.01	0.95	29.17	1.32	0.73
AT7	0.89	1.02	0.94	26.83	1.21	0.60
AT8	0.73	1.11	0.78	29.93	1.23	0.73
AT12-S	0.73	1.11	0.78	31.79	1.14	1.16
AT12-R	0.93	1.03	0.94	25.24	1.18	0.59
AT35	0.90	1.08	0.84	30.08	1.05	0.91
AT15-b	0.83	1.04	0.89	27.72	1.23	0.67
AT17	0.85	1.03	0.92	27.60	1.26	0.72
AT19	0.81	1.09	0.83	28.34	1.13	0.99
AT21	0.87	1.06	0.86	24.69	1.06	0.82
AT43	0.87	1.04	0.92	25.60	1.17	0.57
AT48	1.04	1.04	0.93	26.51	1.29	1.23
AT37	0.87	1.03	0.93	24.57	1.21	0.65
AT45-S	0.95	1.05	0.91	25.41	1.21	0.64
AT46	0.95	1.03	0.93	27.34	1.29	1.73
AT47	0.97	1.07	0.87	27.66	1.31	1.28
AT27	0.91	1.05	0.91	26.38	1.21	0.86
AT28	0.87	1.08	0.85	27.19	1.27	0.65
Average	0.86	1.06	0.87	27.75	1.23	0.89

Supplementary Table 4 Rare Earth Elements and Yttrium data. Post-Archean average Australian Shale (PAAS: Taylor and Mclennan[20])

	La	Ce	Pr	Nd	Sm	Eu	Gd	Tb	Dy	Y	Ho	Er	Tm	Yb	Lu	∑REE
	μg/g	μg/g	μg/g	μg/g	μg/g	μg/g	μg/g	μg/g	μg/g	μg/g	μg/g	μg/g	μg/g	μg/g	μg/g	μg/g
AT2	6.63	11.10	1.61	6.73	1.50	0.39	1.18	0.15	0.89	5.53	0.19	0.55	0.09	0.60	0.10	37.29
AT3-C	7.08	14.40	1.99	8.56	1.97	0.51	1.44	0.19	1.07	5.72	0.21	0.58	0.09	0.57	0.09	44.47
AT4	6.00	10.20	1.39	5.43	1.09	0.26	0.91	0.13	0.76	4.68	0.16	0.46	0.07	0.48	0.08	32.10
AT5-V	1.36	1.99	0.35	1.52	0.32	0.07	0.26	0.03	0.19	1.27	0.04	0.10	0.01	0.09	0.02	7.63
AT5-M	4.27	7.99	1.11	4.51	0.91	0.22	0.73	0.10	0.64	3.83	0.14	0.40	0.06	0.43	0.07	25.40
AT6	3.67	6.70	0.92	4.11	0.95	0.24	0.80	0.12	0.71	4.23	0.15	0.40	0.06	0.40	0.06	23.52
AT7	6.75	12.90	1.66	6.99	1.63	0.39	1.42	0.22	1.37	8.09	0.30	0.86	0.13	0.88	0.14	43.73
AT8	4.72	7.16	1.07	4.35	0.93	0.22	0.79	0.12	0.73	4.80	0.16	0.45	0.07	0.47	0.08	26.14
AT12-S	3.21	4.85	0.72	2.92	0.58	0.13	0.50	0.07	0.42	2.79	0.09	0.23	0.03	0.20	0.03	16.76
AT12-R	12.90	23.60	2.65	9.70	2.04	0.48	1.76	0.30	1.98	11.10	0.44	1.31	0.21	1.43	0.22	70.08
AT35	3.73	7.56	1.00	3.88	0.69	0.14	0.61	0.09	0.59	3.80	0.13	0.35	0.05	0.35	0.05	23.05
AT15-b	4.82	9.10	1.30	5.77	1.26	0.31	1.11	0.17	1.04	6.37	0.23	0.62	0.10	0.62	0.10	32.91
AT17	5.57	10.60	1.50	6.67	1.92	0.49	1.73	0.26	1.54	8.08	0.29	0.74	0.11	0.66	0.10	40.31
AT19	4.87	8.08	1.09	4.23	0.74	0.17	0.64	0.10	0.61	3.65	0.13	0.35	0.05	0.35	0.06	25.11
AT21	10.40	21.80	3.12	13.30	3.52	0.72	2.94	0.44	2.50	11.70	0.48	1.22	0.19	1.21	0.19	73.75
AT43	16.90	29.10	3.56	13.80	3.02	0.73	2.86	0.49	3.15	17.20	0.67	1.88	0.29	1.98	0.31	95.95
AT48	21.40	52.40	6.24	24.00	4.76	1.17	3.83	0.55	3.08	15.90	0.60	1.60	0.25	1.62	0.26	137.64
AT37	3.87	7.00	0.90	3.70	0.90	0.22	0.80	0.12	0.75	3.79	0.15	0.42	0.07	0.45	0.07	23.21
AT45-S	12.20	25.60	3.17	12.40	2.70	0.64	2.28	0.37	2.37	13.10	0.52	1.48	0.24	1.59	0.25	78.93
AT46	20.30	41.60	5.03	19.60	3.91	0.97	3.22	0.41	2.16	11.20	0.41	1.02	0.15	0.93	0.14	111.16
AT47	23.00	54.10	7.08	27.80	5.70	1.47	4.89	0.70	3.71	19.50	0.71	1.84	0.27	1.76	0.27	152.77
AT27	12.30	24.80	3.16	12.70	2.67	0.61	2.09	0.33	1.98	10.80	0.41	1.13	0.18	1.17	0.18	74.50
AT28	14.50	27.80	3.72	14.70	2.65	0.69	2.48	0.38	2.59	16.70	0.61	1.77	0.28	1.83	0.29	91.05
PAAS	38.20	79.60	8.83	33.90	5.55	1.08	4.66	0.77	4.68	27.00	0.99	2.85	0.41	2.82	0.43	211.77

Supplementary Table 5 Enrichment factors of Arsenic and redox sensitive trace elements, and Cu/Zn ratios.

	EF As	EF Co	EF Cr	EF Cu	EF Mo	EF Ni	EF Th	EF U	EF V	EF Zn	EF Cd	EF (P)	Cu/Zn
AT2	391.76	0.86	0.89	2.41	5.12	0.53	0.34	9.76	0.59	1.53	59.08	<L.D.	0.58
AT3-C	628.76	1.33	3.27	26.10	68.29	0.79	0.35	12.72	1.00	6.21	41.88	<L.D.	1.54
AT4	435.30	1.10	0.80	1.79	<L.D.	0.64	0.46	14.13	0.68	1.19	54.84	<L.D.	0.55
AT5-V	14619.44	0.65	2.20	98.95	<L.D.	<L.D.	0.32	154.69	1.86	22.53	1105.85	<L.D.	1.61
AT5-M	666.29	0.73	0.71	2.28	<L.D.	0.43	0.39	12.14	1.45	1.01	37.26	<L.D.	0.83
AT6	593.44	1.49	1.03	2.54	<L.D.	0.70	0.36	11.57	0.77	1.69	48.79	<L.D.	0.55
AT7	211.21	1.33	1.43	0.57	<L.D.	1.01	0.52	16.73	1.09	1.36	21.29	<L.D.	0.15
AT8	364.95	0.96	1.75	<L.D.	<L.D.	0.72	0.47	19.07	0.83	1.39	67.38	<L.D.	<L.D.
AT12-S	2656.91	0.74	1.35	20.55	16.93	<L.D.	0.39	58.44	0.88	2.98	299.14	<L.D.	2.52
AT12-R	50.60	0.75	1.15	0.88	<L.D.	0.60	0.58	11.51	0.84	0.69	1.65	<L.D.	0.47
AT35	778.15	1.02	1.07	43.01	13.11	0.50	0.47	9.46	0.80	1.80	120.15	<L.D.	8.74
AT15-b	260.07	1.39	1.02	1.81	<L.D.	0.64	0.33	7.96	0.75	1.62	30.88	<L.D.	0.41
AT17	572.36	0.75	1.30	0.34	<L.D.	0.62	0.41	11.88	1.21	0.73	23.91	<L.D.	0.17
AT19	917.69	1.43	1.18	6.53	<L.D.	1.19	0.58	12.52	0.66	1.22	63.40	<L.D.	1.97
AT21	62.93	1.22	0.96	0.46	<L.D.	0.73	0.65	6.04	0.89	1.09	1.57	1.77	0.16
AT43	23.80	0.78	0.88	1.43	<L.D.	0.86	0.74	4.50	1.01	2.16	0.65	1.57	0.24
AT48	26.23	0.52	1.18	0.22	<L.D.	0.39	0.56	2.25	0.57	0.98	0.65	1.34	0.08
AT37	67.02	0.94	1.08	0.85	<L.D.	0.73	0.67	13.44	1.02	2.74	1.33	<L.D.	0.11
AT45-S	90.24	1.03	1.44	0.28	<L.D.	0.55	0.77	23.26	0.71	1.94	1.02	1.74	0.05
AT46	74.23	1.05	1.06	3.00	<L.D.	0.72	0.56	13.57	0.71	1.69	1.63	<L.D.	0.65
AT47	32.03	0.67	1.10	0.22	<L.D.	0.49	0.50	3.18	0.68	1.50	0.81	1.32	0.05
AT27	66.91	0.86	0.66	0.57	13.54	0.48	0.70	2.04	1.42	1.38	0.40	1.37	0.15
AT28	53.75	0.69	0.46	0.35	<L.D.	0.33	0.59	1.47	0.82	1.00	0.33	1.05	0.13

Supplementary Table 6 Data of the different salinity proxies (wei and Algeo[31]), compared with data of the Triassic Zhangjiatan (from li et al.,[32].

Formation	Sample	Sr (ppm)	Ba (ppm)	Ga (ppm)	B (ppm)	K%	Sr/Ba	B/K	B/Ga
Triassic Zhangjiatan formation	ZK702H1	163.3	546.3	18.7	21.06	2.46	0.30	8.57	1.13
Triassic Zhangjiatan formation	ZK702H6	110.4	380.9	22.1	22.81	3	0.29	7.61	1.03
Triassic Zhangjiatan formation	ZK702H8	117.2	536.4	17.6	15.21	2.52	0.22	6.03	0.86
Triassic Zhangjiatan formation	ZK702H14	162	515.7	23.7	32.08	1.87	0.31	17.18	1.35
Triassic Zhangjiatan formation	ZK702H18	229.8	463.4	19.2	15.51	1.54	0.50	10.1	0.81
Triassic Zhangjiatan formation	ZK702H24	384.9	774.6	22.1	14.7	1.24	0.50	11.88	0.67
Triassic Zhangjiatan formation	ZK702H32	328.2	792.6	24.1	19.57	1.97	0.41	9.95	0.81
Triassic Zhangjiatan formation	ZK702H35	506.5	830.6	18.5	18.91	3.41	0.61	5.54	1.02
Triassic Zhangjiatan formation	ZK1501H1	137.2	462.1	17.1	14.5	2.41	0.30	6.02	0.85
Triassic Zhangjiatan formation	ZK1501H3	185	562.4	21	18.44	1.76	0.33	10.48	0.88
Triassic Zhangjiatan formation	ZK1501H6	151.7	438.5	22	20.86	1.58	0.35	13.23	0.95
Triassic Zhangjiatan formation	ZK1501H11	438.8	765.1	22.5	20	1.69	0.57	11.87	0.89
Triassic Zhangjiatan formation	ZK1501H15	425	849.8	22.7	26.56	1.96	0.50	13.56	1.17
Triassic Zhangjiatan formation	ZK2709H3	192.4	649.5	19	29.5	2.1	0.30	14.05	1.55
Triassic Zhangjiatan formation	ZK2709H6	182	612	17.9	43.8	1.85	0.30	23.66	2.45
Triassic Zhangjiatan formation	ZK2709H8	177.7	662	20.5	51	2.32	0.27	21.94	2.49
Ediacaran Amane Tazgat formation	AT2	189.98	162.76	4.67	17.5	0.56	1.17	31.42	3.75
Ediacaran Amane Tazgat formation	AT3-C	228.87	222.03	5.53	26.7	0.76	1.03	35.15	4.83
Ediacaran Amane Tazgat formation	AT4	198.19	353.83	4.97	26.2	0.65	0.56	40.41	5.27
Ediacaran Amane Tazgat formation	AT5-V	213.31	16.06	0.2	19.5	<L.D	13.28	<L.D	97.50
Ediacaran Amane Tazgat formation	AT5-M	259.07	54.85	4.63	30.3	0.6	4.72	50.48	6.54
Ediacaran Amane Tazgat formation	AT6	199.27	92.15	3.88	19.7	0.4	2.16	49.44	5.08
Ediacaran Amane Tazgat formation	AT7	138.2	85.47	6.21	11.2	0.55	1.62	20.23	1.80
Ediacaran Amane Tazgat formation	AT8	150.88	47.38	3.6	11.8	0.43	3.18	27.6	3.28
Ediacaran Amane Tazgat formation	AT12-S	223.98	59.37	0.9	20.9	0.13	3.77	160.36	23.22
Ediacaran Amane Tazgat formation	AT12-R	54.08	270.49	11.99	35.6	2.08	0.20	17.09	2.97
Ediacaran Amane Tazgat formation	AT35	185.77	27.37	1.79	12.3	0.06	6.79	189.96	6.87
Ediacaran Amane Tazgat formation	AT15-b	188.88	88.78	5.25	18	0.48	2.13	37.26	3.43
Ediacaran Amane Tazgat formation	AT17	184.41	48.53	4.27	12.9	0.43	3.80	30.23	3.02

AT19	191.05	52.97	1.99	16.7	0.23	3.61	71.34	8.39
AT21	53.9	296.25	10.74	26.2	1.78	0.18	14.76	2.44
AT43	199.96	679.35	19.13	27.1	2.17	0.29	12.51	1.42
AT48	309.85	510.9	13.22	16.5	1.08	0.61	15.22	1.25
AT37	34.07	189.48	4.9	20	0.52	0.18	38.8	4.08
AT45-S	42.75	112.66	10.99	12.5	0.44	0.38	28.68	1.14
AT46	114.24	180.62	8.39	7.4	0.73	0.63	10.19	0.88
AT47	328	705	14.2	7.9	1.36	0.47	5.8	0.56
AT27	135.58	821.37	10.59	11.7	1.48	0.17	7.93	1.10
AT28	237.82	1455.66	12.29	6.9	2.94	0.16	2.34	0.56

Supplementary Table 7 Data of the chemical index of alteration calculated from the equation of Fedo et al.,[25], and corrected from the equation of Panahi et al[26]

Samples	CaO´	CaO*	CIA	CIA Corr
AT2	39.98	1.17	56.92	47.87
AT3-C	38.53	0.91	60.35	54.99
AT4	41.07	0.90	60.01	53.55
AT5-V	52.12	0.10	48.70	34.22
AT5-M	38.49	1.13	56.13	47.72
AT6	42.84	0.95	59.55	49.84
AT7	32.80	1.85	57.55	46.42
AT8	38.55	0.76	59.55	51.47
AT12-S	48.00	0.16	59.30	53.72
AT12-R	3.74	1.86	61.07	58.23
AT35	46.55	0.89	48.50	34.99
AT15-b	35.09	1.63	58.01	46.84
AT17	33.15	2.02	51.85	39.83
AT19	40.21	0.84	51.96	40.77
AT21	0.72	0.72	64.94	66.42
AT43	0.10	0.10	66.04	63.89
AT48	0.28	0.28	63.75	54.09
AT37	0.33	0.33	67.10	63.34
AT45-S	2.24	2.24	58.63	45.89
AT46	24.80	2.30	53.70	42.86
AT47	6.57	5.72	50.55	38.94
AT27	0.11	0.11	65.15	60.68
AT28	0.18	0.18	62.63	61.20
Aver. Of Ca	40.57	1.02	56.03	47,17
Aver. Of Ep	3.91	1.39	61.35	55,25

Supplementary Table 8 Pearson's correlation coefficient (r) between redox sensitive elements and Al for carbonate samples. Values in bold are different from 0 at significance level alpha=0.05

Variables	Co	Cr	Cu	Ni	Th	U	V	Zn	Al2O3
Co	**1**								
Cr	**0.742**	**1**							
Cu	0.059	0.355	**1**						
Ni	**0.927**	**0.785**	0.133	**1**					
Th	**0.749**	**0.683**	0.207	**0.880**	**1**				
U	**0.694**	**0.701**	0.271	**0.847**	**0.891**	**1**			
V	**0.748**	**0.729**	0.151	**0.841**	**0.864**	**0.835**	**1**		
Zn	0.452	**0.772**	**0.704**	0.345	0.135	0.197	0.261	**1**	
Al2O3	**0.855**	**0.745**	0.174	**0.904**	**0.953**	**0.840**	**0.914**	0.266	**1**

Supplementary Table 9 Pearson's correlation coefficient (r) between redox sensitive trace elements and Al for epiclastic samples. Values in bold are different from 0 at significance level alpha=0.05

Variables	Co	Cr	Cu	Ni	Th	U	V	Zn	Al2O3
Co	**1**								
Cr	0.337	**1**							
Cu	0.139	0.058	**1**						
Ni	0.632	0.431	0.576	**1**					
Th	**0.787**	0.582	0.084	0.690	**1**				
U	0.056	0.286	0.011	0.047	0.107	**1**			
V	0.686	0.124	0.135	0.564	**0.815**	0.420	**1**		
Zn	0.504	0.448	0.469	**0.860**	**0.754**	0.156	0.585	**1**	
Al2O3	**0.690**	0.596	0.025	0.533	**0.937**	0.280	**0.725**	0.550	**1**

Supplementary captions

Supplementary Figure 1 Plots of major elements vs Al2O3 %.. 6

Supplementary Figure 2 a Thin section photomicrograph of a stromatolite composed of the alternation of iron crust and grain-sized laminae. b Raman spectra of the marked red point in (a). c Thin section photomicrograph of a stromatolite composed of iron laminae, sparitic crusts and grain-sized microbial laminae. d Raman spectra of the marked red point in (c). 6

Supplementary Figure 3 Th/Sc versus La/Sc diagram demonstrating a mafic provenance of Amane Tazgart samples. Note: Andesite, basalt amd granite values are from Condie[27].......... 7

Supplementary Figure 4 a Al versus Eu/Eu* showing no correlation. b Y/Ho versus Eu/Eu*. 7

Supplementary Figure 5 Pr/Yb_{PAAS} diagram discriminating between samples enriched with LREE ($Pr/Yb_{PAAS}>1$) and those enriched with HREE ($Pr/Yb_{PAAS}<1$)...................................... 7

Supplementary Figure 6 PAAS normalized REE+Y spectra of the averages of carbonates and epiclastic samples, compared with REE+Y spectra of Josefsdal chert. 8

Supplementary Figure 7 Figure IV. 9 (a) Plots of Ce/Ce* versus Eu/Eu* (Shields and Stille [28]) 8

Supplementary Figure 8 Biplots of Fe_2O_3% and P_2O_5% showing no correlation for carbonate samples and a positive correlation for epiclastic samples... 8

Supplementary Figure 9 a Simplified geological map of the Anti-atlas of Morocco. b Structural Map of Oued Dar'a caldera showing the localization of the studied area (modified from Chraiki et al.,[18]). 9

Supplementary Table 1 Major element data 10

Supplementary Table 2 Trace elements Data.. 12

Supplementary Table 3 Data of Cerium anomalies (Ce/Ce*and Ce/Ce* Eq 2) calculated using the equation of Bau and Dulski[21] and Lawrence et al., [29],respectively, Praseodymium anomalies (Pr/Pr*) calculated using the equation of Bau and Dulsk[21], Europium anomalies (Eu/Eu*), Y/Ho and Pr/Yb ratios. Note : REY data were normalized to Post Archean Average Shale (PAAS). 15

Supplementary Table 4 Rare Earth Elements and Ytrium data. Post-Archean average Australian Shale (PAAS: Taylor and Mclennan[20]) 16

Supplementary Table 5 Enrichment factors of Arsenic and redox sensitive trace elements, and Cu/Zn ratios. 17

Supplementary Table 6 Data of the different salinity proxies (wei and Algeo[30]), compared with data of the Triassic Zhangjiatan (from li et al.,[31]. 18

Supplementary Table 7 Data of the chemical index of alteration calculated from the equation of Fedo et al.,[25], and corrected from the equation of Panahi et al[26] 20

Supplementary Table 8 Pearson's correlation coefficient (r) between redox sensitive elements and Al for carbonate samples. Values in bold are different from 0 at significance level alpha=0.05.. 21

Supplementary Table 9 Pearson's correlation coefficient (r) between redox sensitive trace elements and Al for epiclastic samples. Values in bold are different from 0 at significance level alpha=0.05 21

Supplementary references

1. McLennan SM. Relationships between the trace element composition of sedimentary rocks and upper continental crust. *Geochemistry, Geophys Geosystems*. 2001;2(4). doi:10.1029/2000GC000109@10.1002/(ISSN)1525-2027.GERM1
2. Choubert G. Histoire géologique du domaine de l’Anti-Atlas. *Notes Mémoires du Serv Géologique du Maroc 100*. 1952:196.
3. Soulaimani A, Burkhard M. The Anti-Atlas chain (Morocco): The southern margin of the Variscan belt along the edge of the West African craton. *Geol Soc Spec Publ*. 2008;297:433-452. doi:10.1144/SP297.20
4. Choubert G. Histoire géologique du Precambrien de l’Anti-Atlas. *Notes Mem Serv Geol Maroc*. 1963:162-352.
5. Hefferan K, Soulaimani A, Samson SD, et al. A reconsideration of Pan African orogenic cycle in the Anti-Atlas Mountains, Morocco. *J African Earth Sci*. 2014;98:34-46. doi:10.1016/J.JAFREARSCI.2014.03.007
6. Gasquet D, Levresse G, Cheilletz A, Azizi-Samir MR, Mouttaqi A. Contribution to a geodynamic reconstruction of the Anti-Atlas (Morocco) during Pan-African times with the emphasis on inversion tectonics and metallogenic activity at the Precambrian–Cambrian transition. *Precambrian Res*. 2005;140(3-4):157-182. doi:10.1016/J.PRECAMRES.2005.06.009
7. Thomas RJ, Fekkak A, Ennih N, Errami E, Loughlin SC. A new lithostratigraphic framework for the Anti-Atlas Orogen , Morocco. 2004;39:217-226. doi:10.1016/j.jafrearsci.2004.07.046
8. Thomas RJ, Chevallier LP, Gresse PG, et al. Precambrian evolution of the Sirwa Window, Anti-Atlas Orogen, Morocco. *Precambrian Res*. 2002;118(1-2):1-57. doi:10.1016/S0301-9268(02)00075-X
9. Walsh GJ, Benziane F, Aleinikoff JN, et al. Neoproterozoic tectonic evolution of the Jebel Saghro and Bou Azzer—El Graara inliers, eastern and central Anti-Atlas, Morocco. *Precambrian Res*. 2012;216-219:23-62. doi:10.1016/J.PRECAMRES.2012.06.010

10. Hawkins, M.P., Beddoe-Stephens, B., Gillespie MR, Loughlin S, et al. Carte géologique du Maroc au 1/50 000, feuille Tiwit. *Notes Mémoires du Serv Géologique du Maroc*. 2001;404.

11. Hindermeyer J, Gauthier H, Destombes J, Choubert G. Carte géologique du Maroc, Jbel Saghro-Dades (Haut Atlas central, sillon sud-atlasique et Anti-Atlas oriental)—Echelle 1/200,000. *Notes Mém Serv Geol Maroc*. 1977.

12. Algeo TJ, Maynard JB. Trace-element behavior and redox facies in core shales of Upper Pennsylvanian Kansas-type cyclothems. *Chem Geol*. 2004;206(3-4):289-318. doi:10.1016/J.CHEMGEO.2003.12.009

13. Tuduri J, Chauvet A, Barbanson L, et al. The jbel saghro au(–ag, cu) and ag–hg metallogenetic province: product of a long-lived ediacaran tectono-magmatic evolution in the moroccan anti-atlas. *Minerals*. 2018;8(12). doi:10.3390/min8120592

14. Walsh GJ, Aleinikoff JN, Benziane F, Yazidi A, Armstrong TR. U–Pb zircon geochronology of the Paleoproterozoic Tagragra de Tata inlier and its Neoproterozoic cover, western Anti-Atlas, Morocco. *Precambrian Res*. 2002;117(1-2):1-20. doi:10.1016/S0301-9268(02)00044-X

15. Álvaro JJ, Ezzouhairi H, Ait Ayad N, Charif A, Solá R, Ribeiro ML. Alkaline lake systems with stromatolitic shorelines in the Ediacaran volcanosedimentary Ouarzazate Supergroup, Anti-Atlas, Morocco. *Precambrian Res*. 2010;179(1-4):22-36. doi:10.1016/j.precamres.2010.02.009

16. Choubert, G., Faure-Muret A. Les corrélations du Précambrien, Anti-Atlas occidental et central. Colloque international sur les corrélations du Précambrien: Agadir – Rabat, 3–23 mai 1970. Livret guide de l'excursion: Anti-Atlas occidental et central. *Notes Mémoires du Serv Géologique du Maroc 229*. 1970:259.

17. Harrison RW, Yazidi A, Benziane F, et al. Carte géologique au 1/50 000, Feuille Tizgui. *Notes Mémoires du Serv Géologique du Maroc*. 2008;470:131.

18. Chraiki I, Bouougri EH, Chi Fru E, et al. A 571 million-year-old alkaline volcanic lake photosynthesizing microbial community, the Anti-atlas, Morocco. *Geobiology*. 2020. doi:10.1111/GBI.12425

19. Álvaro JJ, González-Acebrón L. Sublacustrine hydrothermal seeps and silicification of

microbial bioherms in the Ediacaran Oued Dar’a caldera, Anti-Atlas, Morocco. *Sedimentology*. 2019;66(6):2048-2071. doi:10.1111/sed.12568

20. Taylor, S R; & McLennan SM. *The Continental Crust: Its Composition and Evolution. United States.* united states; 1985. https://www.osti.gov/biblio/6582885.

21. Bau M, Dulski P. Distribution of yttrium and rare-earth elements in the Penge and Kuruman iron-formations, Transvaal Supergroup, South Africa. *Precambrian Res*. 1996;79(1-2):37-55. doi:10.1016/0301-9268(95)00087-9

22. Lawrence MG, Greig A, Collerson KD, Kamber BS. Rare earth element and yttrium variability in South East Queensland waterways. *Aquat Geochemistry*. 2006;12(1):39-72. doi:10.1007/s10498-005-4471-8

23. Tribovillard N, Algeo TJ, Lyons T, Riboulleau A. Trace metals as paleoredox and paleoproductivity proxies: An update. *Chem Geol*. 2006;232(1-2):12-32. doi:10.1016/j.chemgeo.2006.02.012

24. Algeo TJ, Tribovillard N. Environmental analysis of paleoceanographic systems based on molybdenum-uranium covariation. *Chem Geol*. 2009;268(3-4):211-225. doi:10.1016/j.chemgeo.2009.09.001

25. Fedo CM, Nesbitt HW, Young GM. Unraveling the Effects of Potassium Metasomatism in Sedimentary-Rocks and Paleosols , with Implications for Paleoweathering Conditions and Provenance Unraveling the effects of potassium metasomatism in sedimentary rocks and paleosols , with implications f. *Geology*. 1995;23(october):921-924. doi:10.1130/0091-7613(1995)023<0921

26. Panahi A, Young GM, Rainbird RH. Behavior of major and trace elements (including REE) during Paleoproterozoic pedogenesis and diagenetic alteration of an Archean granite near Ville Marie, Québec, Canada. *Geochim Cosmochim Acta*. 2000;64(13):2199-2220. doi:10.1016/S0016-7037(99)00420-2

27. Kent C. Condie. Chemical composition and evolution of the upper continental crust: Contrasting results from surface samples and shales. *Chem Geol*. 1993;104:1-37.

28. Ward JF, Verdel C, Campbell MJ, Leonard N, Duc Nguyen A. Rare earth element geochemistry of Australian Neoproterozoic carbonate: Constraints on the Neoproterozoic oxygenation events. *Precambrian Res*. 2019;335:105471.

doi:10.1016/j.precamres.2019.105471

29. Shields G, Stille P. Diagenetic constraints on the use of cerium anomalies as palaeoseawater redox proxies: An isotopic and REE study of Cambrian phosphorites. *Chem Geol*. 2001;175(1-2):29-48. doi:10.1016/S0009-2541(00)00362-4

30. Lawrence MG, Kamber BS. The behaviour of the rare earth elements during estuarine mixing-revisited. *Mar Chem*. 2006;100(1-2):147-161. doi:10.1016/j.marchem.2005.11.007

31. Wei W, Algeo TJ. Elemental proxies for paleosalinity analysis of ancient shales and mudrocks. *Geochim Cosmochim Acta*. 2020;287:341-366. doi:10.1016/j.gca.2019.06.034

32. Li D, Li R, Zhu Z, et al. Elemental characteristics and paleoenvironment reconstruction: a case study of the Triassic lacustrine Zhangjiatan oil shale, southern Ordos Basin, China. *Acta Geochim*. 2018;37(1):134-150. doi:10.1007/s11631-017-0193-z
